# QDataSet, quantum datasets for machine learning

**DOI:** 10.1038/s41597-022-01639-1

**Published:** 2022-09-23

**Authors:** Elija Perrier, Akram Youssry, Chris Ferrie

**Affiliations:** 1 Centre for Quantum Software and Information, University of Technology, Sydney, Sydney, 2000 Australia; 2 University of Sydney, Sydney, 2000 Australia; 3 Quantum Photonics Laboratory and Centre for Quantum Computation and Communication Technology, RMIT University, Melbourne, VIC 3000 Australia

**Keywords:** Quantum information, Computer science

## Abstract

The availability of large-scale datasets on which to train, benchmark and test algorithms has been central to the rapid development of machine learning as a discipline. Despite considerable advancements, the field of quantum machine learning has thus far lacked a set of comprehensive large-scale datasets upon which to benchmark the development of algorithms for use in applied and theoretical quantum settings. In this paper, we introduce such a dataset, the QDataSet, a quantum dataset designed specifically to facilitate the training and development of quantum machine learning algorithms. The QDataSet comprises 52 high-quality publicly available datasets derived from simulations of one- and two-qubit systems evolving in the presence and/or absence of noise. The datasets are structured to provide a wealth of information to enable machine learning practitioners to use the QDataSet to solve problems in applied quantum computation, such as quantum control, quantum spectroscopy and tomography. Accompanying the datasets on the associated GitHub repository are a set of workbooks demonstrating the use of the QDataSet in a range of optimisation contexts.

## Abstract (summary)

**Table:** 

Measurement(s)	Simulations of one- and two-qubit quantum systems evolving in the presence and absence of noise and distortion
Technology Type(s)	Simulated measurement using Python packages
Sample Characteristic - Organism	Simulated quantum systems
Sample Characteristic - Environment	Quantum systems in noisy and noiseless environments

## Background & Summary

Quantum machine learning (**QML**) is an emergent multi-disciplinary field combining techniques from quantum information processing, machine learning and optimisation to solve problems relevant to quantum computation^1–4^. The last decade in particular has seen an acceleration and diversification of QML across a rich variety of domains. As a discipline at the interface of classical and quantum computing, subdisciplines of QML can usefully be characterised by where they lie on the classical-quantum spectrum of computation^5^, ranging from quantum-native (using only quantum information processing) and classical (using only classical information processing) to hybrid quantum-classical (a combination of both quantum and classical). At the conceptual core of QML is the nature of how quantum or hybrid classical-quantum systems can *learn* in order to solve or improve results in constrained optimisation problems. The type of machine learning of relevance to QML algorithms very much depends on the specific architectures adopted. This is particularly the case for the use of QML to solve important problems in quantum control, quantum tomography and quantum noise mitigation. Thus QML combines concepts and techniques from quantum computation and classical machine learning, while also exploring novel *quantum* learning architectures.

While quantum-native QML is a burgeoning and important field, the more commonplace synthesis of machine learning concepts with quantum systems arises in classical-quantum hybrid architectures^1,6–8^. Such architectures are typically characterised by a classical parametrisation of quantum systems or degrees of freedom (measurement distributions or expectation values) which are updated according to classical optimisation routine. In applied laboratory and experimental settings, hybrid quantum-classical architectures remain the norm primarily due the fact that most quantum systems rely upon classical controls^9,10^. To this end, hybrid classical-quantum QML architectures which are able to optimise classical controls or inputs for quantum systems have wider, more near-term applicability for both experiments and NISQ^11,12^ devices. Recent literature on hybrid classical-quantum algorithms for quantum control^13,14^, noisy control^15^ and noise characterisation^13^ present examples of this approach. Other recent approaches include the hybrid use of quantum algorithms and classical objective functions for natural language processing^16^. Thus the search for optimising classical-quantum QML architectures is well-motivated from a theoretical and applied perspective.

Despite the increasing maturity of hybrid classical-quantum QML as a discipline, the field lacks many of the characteristics that have been core to the extraordinary successes of classical machine learning in general and deep learning in particular. Classical machine learning has been driven to a considerable extent by the availability of large-scale, high-quality accessible datasets against which algorithms can be developed and tested for accuracy, reliability and scalability. The availability of such datasets as MNIST^17^, ImageNet^18^, Netflix^19^ and other large scale corpora has acted as a catalyst to not just innovations within the machine learning community, but also for the development of benchmarks and protocols that have helped guide the field. Such datasets have also fostered important cross-collaborations among disciplines in ways that have advanced classical machine learning. By contrast, QML as a discipline lacks a similarly standardised set of canonical large-scale datasets against which machine learning researchers (along the quantum-classical spectrum) may benchmark their algorithms and upon which to base innovation. Moreover, the absence of such large-scale standardised datasets arguably holds back important opportunities for cross-collaboration among quantum physicists, computer science and other fields.

In this paper, we seek to address this gap in QML research by presenting a comprehensive dataset for use in QML tailored to solving problems in quantum control, quantum tomography and noise mitigation. The QDataSet is a dedicated resource designed for researchers across classical and quantum computation to develop and train hybrid classical-quantum algorithms for use in theoretical and applied settings relating to these subfields of QML related to control, tomography and noise characterisation of quantum systems. We name this dataset *QDataSet* and our project the *QML Dataset Project*. The motivation behind the QML Dataset Project is to map out a similar data architecture for the training and development of QML as exists for classical machine learning. The focus of the QDataSet on quantum control, tomography and noise problems means that it is of most relevance to these areas as distinct from other areas of QML, such as the development of quantum-only machine learning architectures per se. The contributions of our paper are as follows:

1. Presentation of QDataSet for quantum machine learning, comprising multiple rich large-scale datasets for use in training classical machine learning algorithms for a variety of quantum information processing tasks including quantum control, quantum tomography, quantum noise spectroscopy and quantum characterisation;
2. Presentation of desiderata of QML datasets in order to facilitate their use by theoretical and, in particular, applied researchers; and
3. Demonstration of using the QDataSet for benchmarking classical and hybrid classical-quantum algorithms for quantum control.

## Methods

### Overview

The QDataSet comprises 52 datasets based on simulations of one- and two-qubit systems evolving in the presence and/or absence of noise subject to a variety of controls. It has been developed to provide a large-scale set of datasets for the training, benchmarking and competitive development of classical and quantum algorithms for common tasks in quantum sciences, including quantum control, quantum tomography and noise spectroscopy. It has been generated using customised code drawing upon base-level Python packages and TensorFlow in order to facilitate interoperability and portability across common machine learning and quantum programming platforms. Each dataset consists of 10,000 samples which in turn comprise a range of data relevant to the training of machine learning algorithms for solving optimisation problems. The data includes a range of information (stored in list, matrix or tensor format) regarding quantum systems and their evolution, such as: quantum state vectors, drift and control Hamiltonians and unitaries, Pauli measurement distributions, time series data, pulse sequence data for square and Gaussian pulses and noise and distortion data. The total compressed size of the QDataSet (using Pickle and zip formats) is around 14TB (uncompressed, several petabytes). Researchers can use the QDataSet in a variety of ways to design algorithms for solving problems in quantum control, quantum tomography and quantum circuit synthesis, together with algorithms focused on classifying or simulating such data. We also provide working examples of how to use the QDataSet in practice and its use in benchmarking certain algorithms. Each part below provides in-depth detail on the QDataSet for researchers who may be unfamiliar with quantum computing, together with specifications for domain experts within quantum engineering, quantum computation and quantum machine learning. The Supplementary Information contains extensive background material for researchers unfamiliar with quantum computation. The Supplementary Information also contains discussions of relevant definitions and concepts and so should be read in conjunction with this paper. We also set out further below examples applications of the QDataSet together with links to corresponding Jupyter notebooks. The notebooks are designed to illustrate basic problem solving in tomography, quantum control and quantum noise spectroscopy using the QDataSet. They are designed to enable machine learning researchers to input their algorithms into the relevant section of the code for testing and experimentation. Machine learning uptake often occurs via adapting example code and so we regard these examples as an important demonstration of the use-case for the QDataSet.

### QDataSet methodological overview

In this section, we provide an overview of the methods according to which the QDataSet was developed. Our exposition includes detail of (i) the Hamiltonians used to generate the dataset, (ii) characteristics of control mechanisms used to control the evolution of the quantum simulations and (ii) measurement procedures by which information is extracted from the evolved quantum simulations. This section should be read in tandem with the Supplementary Materials. We aim to equip classical machine learning practitioners with a minimum working knowledge of our methodology to enable them to understand both how the datasets were generated and the ways in which quantum data and quantum computing differ from their classical counterparts relevant to solving problems in quantum control, tomography and noise spectroscopy. Our focus, as throughout this paper, is on the application of classical and hybrid classical-quantum machine learning algorithms and techniques to solve constrained optimisation problems using quantum data (as distinct from the application of purely quantum algorithms). A synopsis of quantum postulates and quantum information processing set out in the Supplementary Material aims also to provide a mapping between ways in which data and computation is characterised in quantum contexts and their analogues in classical machine learning. For example, the description of dataset characteristics, dimensionality, input features, training data to what constitutes labels, the types of loss functions applicable are all important considerations in classical contexts. By providing a straightforward way to translate quantum dataset characteristics into typical classical taxonomies used in machine learning, we aim to help lower barriers to more machine learning practitioners becoming involved in the field of QML. A high-level summary of some of the types of quantum features that QML-dedicated datasets ideally would contain is set out in the Supplementary Information. Our explication of the QDataSet below provides considerable detail on each quantum data feature contained within the datasets including parameters, assumptions behind our choice of quantum system specifications, measurement protocols and noise context. A dictionary of specifications for each example in the QDataSet is set out in Tables (7 and 8).

#### Scalability

The QDataSet was based on simulations of one and two-qubit systems only. The QDataSet was generated over a six-month period using the University of Technology, Sydney’s High Performance Computing (**HPC**) cluster. To generate the datasets, we wrote bespoke code in TensorFlow which enabled us to leverage GPU resources in a more efficient manner. As we discuss below, we were interested in developing a dataset that simulated noise affecting a quantum system. This required performing Monte Carlo simulations and solving Schrödinger’s equation several times for each dataset. While existing packages, such as Qutip (see below) are available to model the effect of noise on quantum systems, we chose not to rely upon such systems. The reason was that Qutip relies upon Lindblad master equations to simulate system/noise interactions which in turn rely upon the validity of certain assumptions and approximations. Chief among these is that noise is Markovian. In our datasets, we included coloured noise with a power-spectrum density which is non-Markovian. Furthermore, Qutip’s methods assumes a model of a quantum system interacting with a fermionic or bosonic bath which was not applicable in our case given we were modelling the imposition of classical noise using Monte Carlo methods.

The resource cost for simulating the various qubit systems depended upon whether we sought to simulate noise or distortion. We found, however, that simulating the two-qubit systems took a significant amount of time, nearly four-weeks of runtime for a single two-qubit system. While multiple nodes of the HPC cluster were utilised, even on the largest node on the cluster (with at least 50–100 cores and two GPUs), the simulation time was extensive, even using GPU-equipped clusters. We estimate that more efficient speedup could be obtained by directly simulating in lower-order languages, such as C++. For this reason, we restricted the QDataSet to simulations of at most two-qubit systems. Such a choice obviously limits direct real-world applications of algorithms trained on the QDataSet to one- and two-qubit systems generally. While this may appear a notable limitation given the growing abundance of higher-order multi-qubit NISQ systems, it remains the case that many experimental laboratories remain limited to small numbers of qubits. We expect in most situations that one and two-qubit gates are all that are available. Engineering more than two-body interactions is an incredible challenge and only available in certain architectures. NISQ devices offer promising next steps, but it is primarily one- and two-qubit systems that have demonstrated the type of long coherence times, fast gate execution and fault-tolerant operation needed for truly scalable quantum computation^11,20,21^. As a result, the QDataSet can be considered relevant to the state of the art. Additionally, simulating more than two qubits would have exceeded computational capacity constraints related to our specific simulated code which includes interactions and iterations over different noise profiles. Moreover, developing algorithms on the basis of small qubit systems is a commonplace way of forming a basis for algorithms for larger multi-qubit systems: training classical machine learning algorithms on lower-order qubit systems has the benefit of enabling researchers to consider how such algorithms can or may learn multi-qubit relations which in turn can assist in algorithm design when applied to higher-order systems. Doing so will be an important step in building scalable databases for applying machine learning to problems in quantum computing.

#### One- and two-qubit hamiltonians

We begin by describing the QDataSet Hamiltonians which are integral to understanding the method by which the datasets were generated. First we describe the single-qubit Hamiltonian and then move to an exposition of the two-qubit case. For the single-qubit system, the drifting Hamiltonian is fixed in the form:

1 $${H}_{d}(t)={H}_{d}=\frac{1}{2}\Omega {\sigma }_{z}.$$

The Ω term represents the energy gap of the quantum system (the difference in energy between, for example, the ground and excited state of the qubit, recalling qubits are characterised by having two distinct quantum states). The single-qubit drift Hamiltonian for the QDataSet is time-independent for simplicity, though in realistic cases it will contain a time-dependent component. For the single-qubit control and noise Hamiltonians we have two cases based upon the concept of which axes controls and noise are applied. Recall we can represent a single qubit system on a Bloch sphere, with axes corresponding to the expectations of each Pauli operator and where operations of each Pauli operator constitute rotations about the respective axis. Our controls are control functions, mostly time-dependent, that apply to each Pauli operator (generator). They act to affect the amplitude over time of rotations about the respective Pauli axes. More detailed treatments of noise in quantum systems and quantum control contexts can be found in ref. ^22^.

As discussed above, the functional form of the control functions *f*_*α*_(*t*) varies. We select both square pulses and Gaussian pulses as the form (see below). Each different noise function *β*_*α*_(*t*) is parameterised differently depending on various assumptions that are more specifically detailed in ref. ^13^ and summarised below. Noise and control functions are applied to different qubit axes in the single-qubit and two-qubit cases. For a single qubit, we first have single-axis control along *x*-direction:

2 $${H}_{{\rm{ctrl}}}(t)=\frac{1}{2}{f}_{x}(t){\sigma }_{x}$$

with the noise (interaction) Hamiltonian *H*_1_(*t*) along *z*-direction (the quantification axis):

3 $${H}_{1}(t)=\frac{1}{2}{\beta }_{z}(t){\sigma }_{z}$$

Here the function *β*_*z*_(*t*) (a classical noise function *β*(*t*) applied along the *z*-axis) may take a variety of forms depending on how the noise was generated (see below for a discussion of noise profiles e.g. N1-N6). It should be noted (for researchers unfamiliar with noise) noise rarely has a functional form and is itself difficult to characterise (so *β*(*t*) should not be thought of as a simple function). For the second case, we implement multi-axis control along *x*- and *y*- directions and noise along x- and *z*-directions in the form:

4 $${H}_{{\rm{ctrl}}}(t)=\frac{1}{2}{f}_{x}(t){\sigma }_{x}+\frac{1}{2}{f}_{y}(t){\sigma }_{y}$$

5 $${H}_{1}(t)=\frac{1}{2}{\beta }_{x}(t){\sigma }_{x}+\frac{1}{2}{\beta }_{z}(t){\sigma }_{z}.$$

Noiseless evolution may be recovered by choosing *H*_1_(*t*) = 0. For the two-qubit system, we chose the drifting Hamiltonian in the form:

6 $${H}_{d}(t)=\frac{1}{2}{\Omega }_{1}{\sigma }_{z}\otimes {\sigma }_{0}+\frac{1}{2}{\Omega }_{2}{\sigma }_{0}\otimes {\sigma }_{z}.$$

For the control Hamiltonians, we also have two cases. The first one is local control along the *x*-axis of each individual qubit, akin to the single-qubit case each. In the notation, *f*_1*α*_(*t*) indicates that the control function is applied to, in this case, the second qubit, while the first qubit remains unaffected (denoted by the ‘1’ in the subscript and by the identity operator *σ*_0_). We also introduce an *interacting control*. This is a control that acts simultaneously on the *x*-axis of each qubit, denoted by *f*_*xx*_(*t*):

7 $${H}_{{\rm{ctrl}}}(t)=\frac{1}{2}{f}_{x1}(t){\sigma }_{x}\otimes {\sigma }_{0}+\frac{1}{2}{f}_{1x}{\sigma }_{0}\otimes {\sigma }_{x}+{f}_{xx}(t){\sigma }_{x}\otimes {\sigma }_{x}.$$

The second two-qubit case is for local-control along the *x*- axis of each qubit only and is represented as:

8 $${H}_{{\rm{ctrl}}}(t)=\frac{1}{2}{f}_{x1}(t){\sigma }_{x}\otimes {\sigma }_{0}+\frac{1}{2}{f}_{1x}{\sigma }_{0}\otimes {\sigma }_{x},$$

For the noise, we fix the Hamiltonian to be along the *z*-axis of both qubits, in the form:

9 $${H}_{1}(t)=\frac{1}{2}{\beta }_{z1}(t){\sigma }_{z}\otimes {\sigma }_{0}+\frac{1}{2}{\beta }_{1z}{\sigma }_{0}\otimes {\sigma }_{z}.$$

Notice, that for the case of local-only control and noiseless evolution, this will correspond to two completely-independent qubits and thus we do not include this case, as it is already covered by the single-qubit datasets. We also note that not all interaction terms (such as *σ*_*z*_ ⊗ *σ*_*z*_ need be included in the Hamiltonian. The reason for this is that to achieve universal control equivalent to including all generators, one only need include one-local control for each qubit together with interacting (entangling) terms. As detailed in the Supplementary Information, assuming one has a minimal set of Pauli generators in the Hamiltonian, one may synthesise any Pauli gate of interest for the one- or two-qubit systems (i.e. given two Pauli gates, one can synthesise the third), thus achieve effective universal control.

To summarise, the QDataSet includes four categories for the datasets set-out in Table 5. The first two categories are for 1-qubit systems, the first is single axis control and noise, while the second is multi-axis control and noise. The third and fourth categories are 2-qubit systems with local-only control or with an additional interacting control together with noise.

#### Control

The control pulse sequences in the QDataSet consist of two types of waveforms. The first is a train of Gaussian pulses, and the other is a train of square pulses, both of which are very common in actual experiments. Square pulses are the simplest waveforms, consisting of a constant amplitude *A*_*k*_ applied for a specific time interval Δ*t*_*k*_:

10 $$f(\Delta {t}_{k})=f={A}_{k}\Delta {t}_{k}$$

where *k* runs over the total number of time-steps in the sequence. The three parameters of such square pulses are the amplitude *A*_*k*_, the position in the sequence *k* and the time duration over which the pulse is applied Δ*t*. In the QDataSet, the pulse parameters are stored in a sequence of vectors {*a*_*n*_}. Each vector *a*_*n*_ is of dimension *r* parameters of each pulse (e.g. the Gaussian pulse vectors store the amplitude, mean and variance, the square pulse vectors store pulse position, time interval and amplitude), enabling reconstruction of each pulse from those parameters if desired. For simplicity, we assume constant time intervals such that Δ*t*_*k*_ = Δ*t*. The Gaussian waveform can be expressed as:

11 $$f(t)=\mathop{\sum }\limits_{k=1}^{n}{A}_{k}{e}^{-{(t-{\mu }_{k})}^{2}/2{\sigma }_{k}^{2}}$$

where *n* is the number of Gaussian pulses in the sequence. The parameters of the Gaussian pulses differ somewhat from those of the square pulses. Each of the *n* pulses in the sequence is characterised by a set of 3 parameters: (i) the amplitude *A*_*k*_ (as with the square pulses), (ii) the mean *μ*_k_ and (iii) the variance *σ*_*k*_ of the pulse sequence. Thus in total, the sequence is characterised by 3*n* parameters. The amplitudes for both Gaussian and square pulses are chosen uniformly at random from the interval [*A*_min_, *A*_max_], the standard deviation for all Gaussian pulses in the train is fixed to *σ*_*k*_ = *σ*, and the means are chosen randomly such that there is minimal amplitude in areas of overlapping Gaussian waveform for the pulses in the sequence. The pulse sequences can be represented in the time or frequency domains^23^. The QDataSet pulse sequences are represented using the time-domain as it has been found to be more efficient feature for machine learning algorithms^13^.

As discussed in^13^, the choice of structure and characteristics of quantum datasets depends upon the particular objectives and uses cases in question, the laboratory quantum control parameters and experimental limitations. Training datasets in machine learning should ideally be structured so as to enhance the generalisability. In the language of statistical learning theory, datasets should be chosen so as to minimise the empirical risk associated with candidate sets of classifiers^24,25^. In a quantum control context, this will include understanding for example the types of controls available to researchers or in experiments, often voltage or (microwave) pulse-based^26^. The temporal spacing and amplitude of each pulse in a sequence of controls applied during an experiment may vary by design or to some degree uncontrollably. Pulse waveforms can also differ. For example, the simplest pulse waveform is a constant-amplitude pulse applied for some time Δ*t*^27^. Such pulses are characterised by for example a single parameter, being the amplitude of the waveform applied to the quantum system (this manifests as we discuss below as an amplitude applied to the algebraic generators of unitary evolution (see refs. ^14,28^ for an example)). Other models of pulses (such as Gaussian) are more complex and require more sophisticated parametrisation and encoding with machine learning architectures in order to simulate. More detail on such considerations and the particular pulse characteristics in the QDataSet are set-out in Tables (7 and 8).

### QDataSet noise methodology

#### Noise characteristics

The QDataSet was developed using methods that aimed to simulate realistic noise profiles in experimental contexts. Noise applicable to quantum systems is generally classified as either *classical* or *quantum*^29^. Classical noise is represented typically as a stochastic process^22^ and can include, for example (i) slow noise which is pseudo-static and not varying much over the characteristic time scale of the quantum system and (ii) fast or ‘white’ noise with a high frequency relative to the characteristic frequencies (energy scales) of the system^30^. The effect of quantum noise on quantum systems is usually classified in two forms. The first is dephasing (*T*_2_) noise, which characteristically causes quantum systems to decohere, thus destroying or degrading quantum information encoded within qubits. Such noise is usually characterised as an operator acting transverse to the quantisation axis of chosen angular momentum.

What this means in practice for the use of the QDataSet is usefully construed as follows using a Bloch sphere. Once an orientation (*x,y,z*-axes) is chosen, one is effectively choosing a choice of basis i.e. the basis of a typical qubit |*ψ*⟩ = *a*|0⟩ + *b*|1⟩ is the basis of eigenstates of the *σ*_*z*_ operator. When noise acts along the *z*-axis (i.e. is associated to the *σ*_*z*_ operator), then it has the potential to (if the energy of the noise is sufficient) shift the energy state in which the quantum system is in, represented by a ‘flip’ in the basis from |0⟩ to |1⟩ for example. This type of noise is *T*_1_ noise. By contrast, noise may act along *x*- and *y*-axes of a qubit, which is represented as being associated with the *σ*_*x*_ and *σ*_*y*_ operators. These axes are ‘transverse’ to the quantisation axis. Noise along these axis has the effect of dephasing a qubit, thus affecting the coherences encoded in the relative phases of the qubit. Such noise is denoted *T*_2_ noise. Open quantum systems’ research and understanding noise in quantum systems is a vast and highly specialised topic. As we describe below, the QDataSet adopts the novel approach outlined in ref. ^13^ where, rather than seeking to fully characterise noise spectra, the only the information about noise relevant to the application of controls (to dampen noise) is sought. Such information is encoded in the *V*_*O*_ operator, which is an expectation that encodes the influence of noise on the quantum system (see the section on “QDataSet noise operators” below). In a quantum control problem using the QDataSet samples containing noise, for example, the objective would then be to select controls that neutralise such effects.

#### QDataSet noise profiles

In developing the QDataSet, we chose sets of noise profiles with different statistical properties. The selected noise profiles have been chosen to emulate commonplace types of noise in experimental settings. Doing so improves the utility of algorithms trained using the QDataSet for application in actual experimental and laboratory settings. While engineers and experimentalists across quantum disciplines will usually be familiar with theoretical and practical aspects of noise in quantum systems, many machine learning and other researchers to whom the QDataSet is directed will not. To assist machine learning practitioners whom may not be familiar with elementary features of noise, it is useful to understand a number of conceptual classifications related to noise used in the QDataSet as follows: (i) power spectral density (which describes the distribution of the noise signal over frequency); (ii) white noise (usually high-frequency noise with a flat frequency); (iii) colored noise, a stochastic process where values are correlated spatially or temporally; (iv) autocorrelated stochasticity, which describes where the noise waveform characteristics are biased by tending to be short (blue) or long (red) as distinct from unautocorrelated noise, where waveforms are relatively uniformly distributed across wavelengths; and (v) stationary noise (a waveform with a constant time period) and non-stationary noise (a waveform with a varying time period). See literature on noise in signal processing for more detail (such as ref. ^31^ for an introduction or ref. ^22^ for a more advanced quantum treatment). The noise realizations are generated in time domain following one of these profile listed as follows (see ref. ^13^ for specific functional forms):

- N0: this is the noiseless case (indicated in the QDataSet parameters as set out in Tables (7 and 8);
- N1: the noise *β*(*t*) is described by its power spectral density (PSD) *S*_1_(*f*), a form of 1/*f* noise with a Gaussian bump;
- N2: here *β*(*t*) is stationary Gaussian colored noise described by its autocorrelation matrix; chosen such that it is colored, Gaussian and stationary (typically lower frequency) and is produced via convolving Gaussian white noise with a deterministic signal;
- N3: here the noise *β*(*t*) is non-stationary Gaussian colored noise, again described by its autocorrelation matrix which is chosen such that it is colored, Gaussian and non-stationary. The noise is simulated via multiplication of a deterministic time-domain signal with stationary noise;
- N4: in this case, the noise *β*(*t*) is described by its autocorrelation matrix chosen such that it is colored, non-Gaussian and non-stationary. The non-Gaussianity of the noise is achieved via squaring the Gaussian noise so as to achieve requisite non-linearities;
- N5: a noise described by its power spectral density (PSD) *S*_5_(*f*), differing from N1 only via the location of the Gaussian bump; and
- N6: this profile is to model a noise source that is correlated to one of the other five sources (N1-N5) through a squaring operation. If the *β*(*t*) is the realization of one of the five profiles, N6 will have realizations of the form *β*^2^(*t*). This profile is used for multi-axis and multi-qubit systems.

The N1 and N5 profiles can be generated following the method described in ref. ^13^ (see the section entitled “Implementation” onwards). Regarding the other profiles, any standard numerical package can generate white Gaussian stationary noise. The QDataSet noise realisations were encoded using the Numpy package of Python. We deliberately did so in order to avoid various assumptions used in common quantum programming packages, such as Qutip.

To add coloring, we convolve the time-domain samples of the noise with some signal. To generate non-stationarity, we multiply the time-domain samples by a some signal. finally, to generate non-Gaussianity, we start with a Gaussian noise and apply non-linear transformation such as squaring. The last noise profile is used to model the case of two noise sources that are correlated with each other. In this case we generate the first one using any of the profiles N1-N5, and the other source is completely determined.

#### Distortion

In physical experiments, the control pulses are physical signals (such as microwave pulses), which propagate along cables and get processed by different devices. This introduces distortions which cannot be avoided in any real devices. However, by properly engineering the systems, the effects of these distortions can be minimized and/or compensated for in the design. In this paper, we used a linear-time invariant system to model distortions of the control pulses, and the same filter is used for all datasets. We chose a Chebychev analogue filter^32^ with an undistorted control signal is the input and distorted the filter output signal. Table (6) sets out a summary of key parameters.

#### QDataSet noise operators

In developing the QDataSet, we have assumed that the environment affecting the qubit is classical and stochastic, namely that *H*_1_(*t*) will be a stochastic term that acts directly on the system. The stochasticity of *H*_1_(*t*) means that the expectation of any observable measured experimentally will be given as:

12 $${O}_{c}={\rm{Tr}}{(\rho (T)O)}_{c},$$

where *O* represents the measurement operator corresponding to the observable of interest (e.g. $O\mathop{=}\limits^{.}{M}_{m}$ in notation above) and the 〈·〉_*c*_ is a classical expectation over the distribution of the noise realizations. It can then be shown (see ref. ^13^) that this can be expressed in term of the initial state *ρ*(0), and the evolution fixed over the time interval [0, *T*] as:

13 $$O(T)={\rm{Tr}}\left({V}_{O}(T){U}_{0}^{\dagger }(T)\rho (0){U}_{0}(T)O\right)$$

where ${U}_{0}(T)={{\mathscr{T}}}_{+}{e}^{-i{\int }_{0}^{T}{H}_{0}(t)dt}$ is the evolution matrix in the absence of noise and:

14 $${V}_{O}(T)={W}_{O}{(T)}_{c}$$

15 $$\mathop{=}\limits^{.}{\left\langle {O}^{-1}{\widetilde{U}}_{I}^{\dagger }(T)O{\widetilde{U}}_{I}(T)\right\rangle }_{c}.$$

is a novel noise operator introduced in^13^ which characterises the expectation of noise relevant to synthesising counteracting control pulses. We encapsulate the full quantum evolution via the operator *W*_*O*_ (*T*). Note that *V*_*O*_ is formed via the partial tracing out of the effect of the noise and its interaction with the control pulses, so encodes only those features of importance or relevance to impact of noise (not the full noise spectrum). Importantly, the use of the *V*_*O*_ operator is designed to allow information about noise and observables to be separated (a hallmark of dynamic decoupling approaches). The modified interaction unitary ${\widetilde{U}}_{I}(T)$ is defined such that:

16 $$U(T)={\widetilde{U}}_{I}(T){U}_{0}(T),$$

where $U(T)={{\mathscr{T}}}_{+}{e}^{-i{\int }_{0}^{T}H(t)dt}$ is the full evolution matrix. This contrasts the conventional definition of the interaction unitary which takes the form *U*(*T*) = *U*_0_(*T*)*U*_*I*_(*T*). The *V*_*O*_ operator is used in the simulation code for the QDataSet to characterise the effect of noise on such values. Ideally, in a noise-free scenario, those expectations should tend to zero (representative of the absence of noise). The idea for including such noise operators is that this data can then be input into machine learning models to assist the algorithms to learn appropriate, for example, pulse sequences or controls that send *V*_*O*_ → *I* (neutralising the noise).

A detailed explanation and example of the use of the *V*_*O*_ operator is provided in ref. ^13^. For machine learning practitioners, the operator *V*_*O*_ may, for example, be used in an algorithm that seeks to negate the effect of *V*_*O*_. The utility of this approach is that full noise spectroscopy is not required.

### QDataSet measurement methodology

#### QDataSet POVMs

The simulated quantum measurements of the QDataSet are inherently probabilistic. As set out in the Supplementary Information, measurement of quantum systems yields an underlying probability distribution over the possible measurement outcomes (observables) *m*_*i*_ of the system which are in turn determined by the state of the system and the measurement process. There are several ways to describe quantum measurements mathematically. The most common method (which we adopted) involves projective measurements. In this case, an observable *O* is described mathematically by a Hermitian operator. The eigendecomposition of the operator can be expressed in the form *O* = ∑_*m*_ *mP*_*m*_, where *m* are the eigenvalues, and *P*_*m*_ are the associated projectors into the corresponding eigenspace. The projectors *P*_*m*_ must satisfy that ${P}_{m}^{2}={P}_{m}$, and that ∑_*m*_ *P*_*m*_ = *I* (the identity operator), to ensure we get a correct distribution for the outcomes. In more sophisticated treatments, the operators *O* belong to POVM described above which partition the Hilbert space ${\mathscr{H}}$ into distinct projective subspaces ${{\mathscr{H}}}_{m}$ associated with each POVM operator *O*. The probability of measuring an observable is given by:

17 $${\rm{\Pr }}(m)={\rm{Tr}}(\rho {P}_{m}),$$

for a system in the state *ρ*. The expectation value of the observable is given by:

18 $$\left\langle O\right\rangle ={\rm{Tr}}(\rho O)={\rm{Tr}}\left(\rho \sum _{m}m{P}_{m}\right)=\sum _{m}m\Pr (m).$$

As detailed below, the QDataSet contains measurement outcomes for a variety of noiseless and noisy systems. The POVM chosen is the set of Pauli operators for one and two-qubit systems. The measurement operators chosen are the Pauli operators described below and the QDataSet contains the expectation values for each Pauli measurement operator. In a classical machine learning context, these measurement statistics form training data labels in optimisation problems, such as designing algorithms that can efficiently sequence control pulses in order to efficiently (time-minimally) synthesise a target state or unitary (and thus undertake a quantum computation) of interest.

#### Pauli matrices

The POVM for the QDataSet is the set of Pauli operators which are important operators in quantum information processing involving qubit systems. This is in part because such qubit systems can be usefully decomposed into a Pauli operator basis via the Pauli matrices:

19 $${\sigma }_{x}=\left(\begin{array}{ll}0 & 1\\ 1 & 0\end{array}\right),{\sigma }_{y}=\left(\begin{array}{ll}0 & -i\\ i & 0\end{array}\right),{\sigma }_{z}=\left(\begin{array}{ll}1 & 0\\ 0 & -1\end{array}\right)$$

together with the identity (denoted *σ*_0_). Pauli operators are Hermitian (with eigenvalues +1 and −1), traceless and satisfy that ${\sigma }_{i}^{2}=I$. Together with the identity matrix (which is sometime denoted by *σ*_0_), they form an orthonormal basis (with respect to the Hilbert-Schmidt product defined as *A*,*B* = Tr(*A*^†^*B*)) for any 2 × 2 Hermitian matrix. QDataSet qubit states can then be expressed in this basis via the density matrix:

20 $$\rho =\frac{1}{2}\left(I+{\bf{r}}\cdot \sigma \right),$$

where the vector **r** = (*r*_*x*_, *r*_*y*_, *r*_*z*_) is a unit vector called the Bloch vector, and the vector *σ* = (*σ*_*x*_, *σ*_*y*_, *σ*_*z*_). The dot product of these two vectors is just a shorthand notation for the expression **r**·*σ* = *r*_*x*_ *σ*_*x*_ + *r*_*y*_ *σ*_*y*_ + *r*_*z*_ *σ*_*z*_. Any time-dependent Hamiltonian of a qubit can be expressed as

21 $$H(t)=\sum _{i\in \{x,y,z\}}{\alpha }_{i}(t){\sigma }_{i},$$

with the time-dependence absorbed in the coefficients *α*_*i*_ (*t*).

#### Pauli measurements

The measurements simulated in the QDataSet are what are known as Pauli measurements. These are formed by taking the expectation value of each Pauli matrix e.g. Tr(*ρσ*_*i*_) for *I* ∈ {*x*, *y*, *z*} (the identity is omitted). The resultant measurement distributions will typically form labelled data in a machine learning context. Measurement distributions are ultimately how various properties of the quantum system are inferred (i.e. via reconstructive inference), such as the characteristics of quantum circuits, evolutionary paths and tomographical quantum state description. As we describe below, measurements in the QDataSet comprise measurements on each eigenstate (six in total) of each Pauli operator by all Pauli operators. Hermitian operators have a spectral decomposition in terms of eigenvalues and their corresponding projectors

22 $${P}_{0}=\left|0\right\rangle \left\langle 0\right|=\frac{1}{2}(I+{\sigma }_{z})=\left(\begin{array}{ll}1 & 0\\ 0 & 0\end{array}\right)$$

23 $${P}_{1}=\left|0\right\rangle \left\langle 0\right|=\frac{1}{2}(I-{\sigma }_{z})=\left(\begin{array}{ll}0 & 0\\ 0 & 1\end{array}\right)$$

thus we can write:

24 $${\sigma }_{z}=1\times \left(\begin{array}{ll}1 & 0\\ 0 & 0\end{array}\right)-1\times \left(\begin{array}{ll}0 & 0\\ 0 & 1\end{array}\right)$$

For example, a Pauli measurement on a qubit in the −1 eigenstate with respect to the *σ*_*z*_ operator

25 $${\rm{Tr}}(\rho {\sigma }_{z}^{\dagger })={\rm{Tr}}\left(\left(\begin{array}{ll}0 & 0\\ 0 & 1\end{array}\right)\left(\begin{array}{ll}1 & 0\\ 0 & -1\end{array}\right)\right)={\rm{Tr}}\left(\begin{array}{ll}0 & 0\\ 0 & -1\end{array}\right)=-1$$

which is as expected. The probability of observing *λ* = −1 in this state we should expect to be unity (given the state is in the eigenstate):

26 $$Pr(m=-1)={\rm{Tr}}\left({P}_{1}^{2}\rho \right)=1$$

For *n*-qubit systems (such as two-qubit systems in the QDataSet), Pauli measurements are represented by tensor-products of Pauli operators. For example, a *σ*_*z*_ measurement on the first qubit and *σ*_*x*_ on the second is represented as:

27 $${\sigma }_{z}\otimes {\sigma }_{x}$$

In programming matrix notation, this becomes represented as a 4 × 4 matrix (tensor):

28 $${\sigma }_{z}\otimes {\sigma }_{X}=\left(\begin{array}{ll}1 & 0\\ 0 & -1\end{array}\right)\otimes \left(\begin{array}{ll}0 & 1\\ 1 & 0\end{array}\right)$$

29 $$=\left(\begin{array}{llllll}1 & \times  & \left(\begin{array}{ll}0 & 1\\ 1 & 0\end{array}\right) &  &  & 0\\  &  & \,0 & -1 & \times  & \left(\begin{array}{ll}0 & 1\\ 1 & 0\end{array}\right)\end{array}\right)$$

30 $$=\left(\begin{array}{llll}0 & 1 & 0 & 0\\ 1 & 0 & 0 & 0\\ 0 & 0 & 0 & -1\\ 0 & 0 & -1 & 0\end{array}\right)$$

The Pauli representation of qubits used in the QDataSet can be usefully visualised via the Bloch sphere as per Fig. 4. The axes of the Bloch sphere are the expectation values of the Pauli *σ*_*x*_, *σ*_*y*_ and *σ*_*z*_ operators respectively. As each Pauli operator has eigenvalues 1 and −1, the eigenvalues can be plotted along axes of the 2-sphere. For a pure (non-decohered) quantum state *ρ*, $| \rho | =\sqrt{{r}_{x}^{2}+{r}_{y}^{2}+{r}_{z}^{2}}=1$ (as we require Tr*ρ*^2^ = 1), thus *ρ* is represented on the Bloch 2-sphere as a vector originating at the origin and lying on the surface of the Bloch 2-sphere. The evolution of the qubit i.e. a computation according to unitary evolution can then be represented as rotations of *ρ* across the Bloch sphere. In noisy contexts, decohered *ρ* are represented whereby |ρ| < 1 i.e. the norm of *ρ* shrinks and *ρ* no longer resides on the surface.

For machine learning practitioners, it is useful to appreciate the operation of the QDataSet Pauli operators *σ*_*x*_, *σ*_*y*_, *σ*_*z*_ as the generators of rotations about the respective axes of the Bloch sphere. Represented on a Bloch sphere, the application of *σ*_*z*_ to a qubit is equivalent to rotating the quantum state vector *ρ* about the z-axis (see Fig. 4). Conceptually, a qubit is in a *z*-eigenstate if it is lying directly on either the north (+1) or south (−1) pole. Rotating about the *z*-axis then is akin to rotating the vector on the spot, thus no change in the quantum states (or eigenvalues) for *σ*_*z*_ occurs because the system exhibits symmetry under such transformations. This is similarly the case for *σ*_*x*_, *σ*_*y*_ generators with respect to their eigenvalues and eigenvectors. However, rotations by *σ*_*α*_ will affect the eigenvalues/vectors in the *σ*_*β*_ basis where *α* ≠ *β* e.g. a *σ*_*x*_ rotation will affect the component of the qubit lying along the *σ*_*z*_ axis. Similarly, a *σ*_*z*_ rotation of a qubit in a *σ*_*x*_ eigenstate will alter that state (shown in (a) and (b) of Fig. 4). An understanding of Pauli operators and conceptualisation of qubit axes is important to the understanding of the simulated QDataSet. An understanding of symmetries of relevance to qubit evolution (and quantum algorithms) is also beneficial. As we describe below, controls or noise are structured to be applied along particular axes of a qubit and thus can be thought of as a way to control or distortions upon the axial rotations of a qubit effected by the corresponding Pauli generator.

There exist higher dimensional generalization to the Pauli matrices that allow forming orthonormal basis to represent operators in these dimensions. In particular if we have a system of *N* qubits, then one simple generalization is to form the set ${\{{\sigma }_{{i}_{1}}^{(1)}\otimes {\sigma }_{{i}_{2}}^{(2)}\otimes \cdots {\sigma }_{{i}_{N}}^{(N)}\}}_{{i}_{j}\in \{0,x,y,z\}}$. In other words we take tensor products of the Pauli’s which gives a set of size 4^*N*^. For example, for a two-qubit system we can form the 16- element set $\{{\sigma }_{0}\otimes {\sigma }_{0},{\sigma }_{0}\otimes {\sigma }_{x},$ ${\sigma }_{0}\otimes {\sigma }_{y},{\sigma }_{0}\otimes {\sigma }_{z},{\sigma }_{x}\otimes {\sigma }_{0},{\sigma }_{x}\otimes {\sigma }_{x},{\sigma }_{x}\otimes {\sigma }_{y},{\sigma }_{x}\otimes {\sigma }_{z},{\sigma }_{y}\otimes {\sigma }_{0},{\sigma }_{y}\otimes {\sigma }_{x},{\sigma }_{y}\otimes {\sigma }_{y},{\sigma }_{y}\otimes {\sigma }_{z},{\sigma }_{z}\otimes {\sigma }_{0},{\sigma }_{z}\otimes {\sigma }_{x},{\sigma }_{z}\otimes {\sigma }_{y},$ ${\sigma }_{z}\otimes {\sigma }_{z}\}.$ Moreover, for many use cases, we are interested in the *minimal* number of operators, such as Pauli operators, required to achieve a requisite level of control, such as universal quantum computation.

For the single qubit system, initial states are the two eigenstates of each Pauli operator. As noted above, the quantum state can be decomposed in the Pauli basis as ${\rho }_{j}=\frac{1}{2}\left(I\pm {\sigma }_{j}\right)$, for *j* = 1, 2, 3. This gives a total of 6 states. We perform the three Pauli measurements on each of these states, resulting in a total of 18 possible combinations. These 18 measurements are important to characterize a qubit system. For two-qubits, it will be similar but now we initialize every individual qubit into the 6 possible eigenstates, and we measure all 15 Pauli observables (we exclude identity). This gives a total of 540 possible combinations.

#### Monte carlo measurements

Measurements of the one- and two-qubit systems for the QDataSet are undertaken using Monte Carlo techniques. This means that a random Pauli measurement is undertaken multiple times, with the measurement results averaged in order to provide the resultant measurement distribution for each of the POVM operators. The measurement of the quantum systems is contingent on the noise realisations for each system. For the noiseless case, the Pauli measurements are simply the Monte Carlo averages (expectations) of the Pauli operators. Systems with noise will have one or more noise realisations (applications of noise) applied to them. To account for this, we include two separate sets of measurement distribution. The first the expectation value of the three Pauli operators over all possible initial states for each different noise realisation. These statistics are given by the set {*V*_*O*_} in the QDataSet. Thus for each type of noise, there will be a set of measurement statistics. The second is a set of measurement statistics where we average over all noise realisations for the dataset. This second set of measurements is given by the set {*E*_*O*_}. Including both sets of measurements enables algorithms trained using the QDataSet to be more fine-grained in their treatment of noise: in some contexts, while noise profiles may be uncertain, it is clear that the noise is of a certain type, so the first set of measurement statistics may be more applicable. For other cases, there is almost no information about noise profiles or their sources, in which case the average over all noise realisations may be more appropriate.

#### Monte carlo simulator

For the benefit of researchers using the QDataSet, we briefly set out a bit more detail of how the datasets were generated. The simulator comprises three main components. The first approximates time-ordered unitary evolution. The second component generates realisations of noise given random parametrisations of the power spectral density (PSD) of the noise. The third component simulates quantum measurement. The simulations are based upon Monte Carlo methods whereby *K* randomised pulse sequences give rise to noise realisations. The quantum systems are then measured to determine the extent to which the noise realisations affect the expectation values. Trial and error indicated a stabilisation of measurement statistics at around *K* = 500, thus *K* ≥ 1000 was chosen for the final simulation run to generate the QDataSet. The final Pauli measurements are then averages over such noise realisations. The parameter *K* is included for each dataset and example (as described below). For more detail, including useful pseudocode that sets out the relationship between noise realisations, *β*(*t*) and measurement, see the Supplementary Material in ref. ^13^.

## Data Records

### Dataset description and format

The QDataSet is available via the Cloudstor online data repository (https://cloudstor.aarnet.edu.au/plus/s/rxYKXBS7Tq0kB8o) as set out in ref. ^33^.

#### QDataSet form

Quantum information in the QDataSet is stored following the Tensorflow convention of interpreting multidimensional arrays. For example the noise Hamiltonian for one example is stored as a (1, *M*, *K*, 2, 2) array, where the first dimension is the batch, the second is time assuming *M* steps, then whatever comes next is related to the object itself. In this case the third dimension denotes the noise realization assuming a maximum of *K* realizations, and the last two dimensions ensure we have a square matrix of size 2.

The simulation of the datasets is based on a Monte Carlo method, where a number of evolution trajectories are simulated and then averaged to calculate the observables. The exact details can be found in ref. ^13^ (see “Implementation” section) which we reproduce and expand upon in this paper for completeness.

#### QDataSet parameters

Further detail regarding the 52 datasets that we present in this paper for use solving engineering applications discussed in section using classical machine learning can be found on the repository for the QDataSet^33^. Table (1) sets out the taxonomy of each of the 52 different datasets. Each dataset comprises 10,000 examples that are compressed into a Pickle file which is in turn compressed into a zip file. The *Item* field indicates the dictionary key and the *Description* field indicates the dictionary value.

**Table 1:** QDataSet characteristics.

Item	Description
*simulation_ parameters*	*name*: name of the dataset; *dim*: the dimension 2^*n*^ of the Hilbert space for *n* qubits (dimension 2 for single qubit, 4 for two qubits); Ω: the spectral energy gap; *static_operators*: a list of matrices representing the time-independent parts of the Hamiltonian (i.e. drift components); *dynamic_operators*: a list of matrices representing the time-dependent parts of the Hamiltonian (i.e. control components), without the pulses. So, if we have a term $f(t){\sigma }_{x}+g(t){\sigma }_{y}$, this list will be $[{\sigma }_{x},{\sigma }_{y}]$. This dynamic operators are further distinguished (and labelled) according to being (i) undistorted pulses (labelled *pluses*) or (ii) distorted pulses (labelled *distorted*); *noise_operators*: a list of time-dependent parts of the Hamiltonian that are stochastic (i.e. noise components). so if we have terms like ${\beta }_{1}(t){\sigma }_{z}+{\beta }_{2}(t){\sigma }_{y}$, the list will be $[{\sigma }_{z},{\sigma }_{y}]$; *measurement_operators*: Pauli operators (including identity) ($I,{\sigma }_{x},{\sigma }_{y},{\sigma }_{z}$)’ *initial_states*: the six eigenstates of the Pauli operators; *T*: total time (normalised to unity); *num_ex*: number of examples, set to 10,000; *batch_size*: size of batch used in data generation (default is 50); *K*: number of randomised pulse sequences in Monte Carlo simulation of noise (set to *K*=2000); *noise_profile*: N0 to N6 (see above); *pulse_shape*: Gaussian or Square; *num_pulses*: number of pulses per interval; *elapsed_time*: time taken to generate the datasets.
*pulse_parameters*	The control pulse sequence parameters for the example: Square pulses: *A*_*k*_ amplitude at time *t*_*k*_; Gaussian pulses: *A*_*k*_ (amplitude), *μ* (mean) and *σ* (standard deviation).
*time_range*	A sequence of time intervals $\Delta {t}_{j}$ with *j* = 1, ..., *M*;
*pulses*	Time-domain waveform of the control pulse sequence.
*distorted_pulses*	Time-domain waveform of the distorted control pulse sequence (if there are no distortions, the waveform will be identical to the undistorted pulses).
*expectations*	The Pauli expectation values 18 or 52 depending on whether one or two qubits (see above). For each state, the order of measurement is: ${\sigma }_{x},{\sigma }_{y},{\sigma }_{z}$ applied to the evolved initial states. As the quantum state is evolving in time, the expectations will range within the interval [1, −1].
*V*_*O*_ *operator*	The *V*_*O*_ operators corresponding to the three Pauli observables, obtained by averaging the operators *W*_*O*_ over all noise realizations.
*noise*	Time domain realisations of the relevant noise.
*H*_0_	The system Hamiltonian *H*_0_(*t*) for time-step *j*.
*H*1	The noise Hamiltonian *H*_1_(*t*) for each noise realization at time-step *j*.
*U*_0_	The system evolution matrix *U*_0_(*t*) in the absence of noise at time-step *j*.
*U*_*I*_	The interaction unitary *U*_*I*_(*t*) for each noise realization at time-step *j*.
*V*_*O*_	Set of 3 × 2000 expectation values (measurements) of the three Pauli observables for all possible states for each noise realization. For each state, the order of measurement is: ${\sigma }_{x},{\sigma }_{y},{\sigma }_{z}$ applied to the evolved initial states.
*E*_*O*_	The expectations values (measurements) of the three Pauli observables for all possible states averaged over all noise realizations. For each state, the order of measurement is: ${\sigma }_{x},{\sigma }_{y},{\sigma }_{z}$ applied to the evolved initial states.

The left column identifies each item in the respective QDataSet examples (expressed as keys in the relevant Python dictionary) while the description column describes each item.

### Datasets and naming convention

Each dataset can be categorised according to the number of qubits in the system and the noise profile to which the system was subject. Table (5) sets out a summary of such categories. While other types of profiles or combinations could have been utilised, our aim was to select categories which reflect the types of noise and categorisations relevant to experimental laboratories working on problems such as quantum computation. For category 1 of the datasets, we created datasets with noise profiles N1, N2, N3, N4, together with the noiseless case. This gives a total of 5 datasets. For category 2, the noise profiles for the X and Z axes respectively are chosen to be (N1, N5), (N1, N6), (N3, N6). Together with the noiseless case, this gives a total of 4 datasets. For category 3 (two-qubit system), we chose only the 1Z (identity on the first qubit, noise along the z- axis for the second) and Z1 (noise along the z- axis for the first qubit, identity along the second) noise to follow the (N1,N6) profile. This category simulates two individual qubits with correlated noise sources. For category 4, we generate the noiseless, (N1, N5), and (N1, N6) for the 1Z and Z1 noise. This gives 3 datasets. Therefore, the total number of datasets at this point is 13. Including the two types of control waveforms, this gives a total of 26. If we also include the cases of distortion and non-distorted control, then this gives a total of 52 datasets. Comprehensive detail on the noise profiles used to generate the datasets is set-out above.

We chose a convention for the naming of the dataset to try delivering as much information as possible about the chosen parameters for this particular dataset. The name is partitioned into 6 parts, separated by an underscore sign “_”. We explicate each part below:

1. The first part is either the letter “G” or “S” to denote whether the control waveform is Gaussian or square.
2. The second part is either “1q” or “2q” to denote the dimensionality of the system (i.e. the number of qubits).
3. The third part denotes the control Hamiltonian. It is formed by listing down the Pauli operators we are using for the control for each qubit, and we separate between qubit by a hyphen “-”. For example, category 1 datasets will have “X”, while category 4 with have “IX-XI-XX”.
4. The fourth part and fifth parts indicate (i) the axis along which noise is applied (fourth part) and (ii) the type of noise along each axis (fifth part). So “G_2q_IX-XI_IZ-ZI_N1-N6” represents two qubits with control along the *x* axis of each qubit, while the noise is applied along the *z*-axis of each. In this case, N1 noise is applied along the *z*-axis of the first qubit and N6 noise is applied along the *z*-axis of the second qubit. For datasets where no noise is applied, these two parts are omitted.
5. Finally, the sixth part denotes the presence of control distortions by the letter “D”, otherwise it is not included.

For example, the dataset “G_2q_IX-XI-XX_IZ-ZI_N1-N6” is two qubit, Gaussian pulses with no distortions, local X control on each qubit and an interacting XX control along with local noise on each qubit with profile N1 on the first qubit *z*-axis and N6 on the second qubit *z*-axis. Another example the dataset “S_1q_XY_D”, is a single-qubit system with square distorted control pulses along X and Y axis, and there is no noise.

## Technical Validation

Technical validation of the QDataSet was undertaken by comparing the QDataSet data against leading quantum simulation toolkit *Qutip*, an open-source software for simulating the dynamics of open quantum systems^34^. For each of the 26 different simulated systems (each comprising a noisy and noise-free case), the procedure set out below was adopted. A Jupyter notebook containing the code used for technical validation and verification of the datasets is available on the QDataSet repository.

### Distortion analysis

**Fig. 1:**
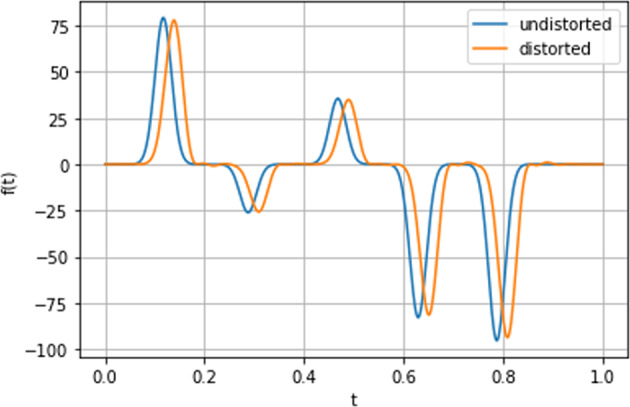
Plot of an undistorted (orange) pulse sequence against a related distorted (blue) pulse sequence for the single-qubit Gaussian pulse dataset with *x*-axis control (‘G_1q_X’) over the course of the experimental runtime. Here *f(t)* is the functional (Gaussian) form of the pulse sequence for time-steps *t*. These plots were used in the first step of the verification process for QDataSet. The shift in pulse sequence is consistent with expected effects of distortion filters. The pulse sequences for each dataset can be found in *simulation_parameters* =⇒ *dynamic_operators* =⇒ *pulses* (undistorted) or *distorted_pulses* for the distorted case (see Table (1) for a description of the dataset characteristics).

**Fig. 2:**
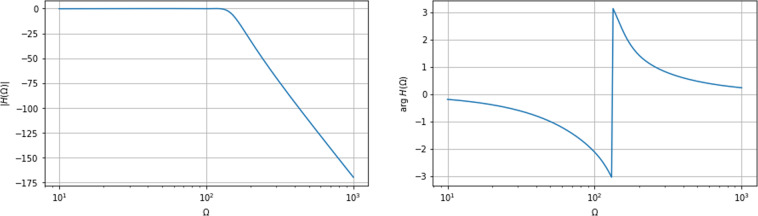
The frequency response (left) and the phase response (right) of the filter that is used to simulate distortions of the control pulses. The frequency is in units of Hz, and the phase response is in units of rad.

Firstly, for each of the one- and two-qubit datasets, the distorted and undistorted pulse sequences were compared for a sample of examples from each dataset in order to assess the effect of relevant distortion filters. A plot of the comparison for the single-qubit case with Gaussian control along the *x*-axis is presented in Fig. (1). The plot compares the distorted sequence of control Gaussian control pulses for the undistorted case (blue) and distorted case (orange). The expectation was for a shift in the Gaussian pulse curves as a result of the distortion filters. An error with the datasets would have seen the distorted pulses not resemble such the undistorted pulses significantly and, moreover, would have likely seen non-Gaussian pulse forms. As can be seen from Fig. (1), the distorted and undistorted pulses appear almost identical but for a shift and a minor amplitude (*f*(*t*)) reduction in the distorted case, which was seen for each example. This provided us with assurance that simulation was appropriately modelling distortion of the pulses. As a second assurance, we plotted the effect of the distortion filter (when applied) and evaluate the frequency response of the filter. The aim of this process was to identify visually whether the form frequency response *H*(Ω) and phase response Ω exhibit the appropriate form (i.e. no obvious errors). The verification plot is shown in Fig. (2).

### Comparison with Qutip

**Fig. 3:**
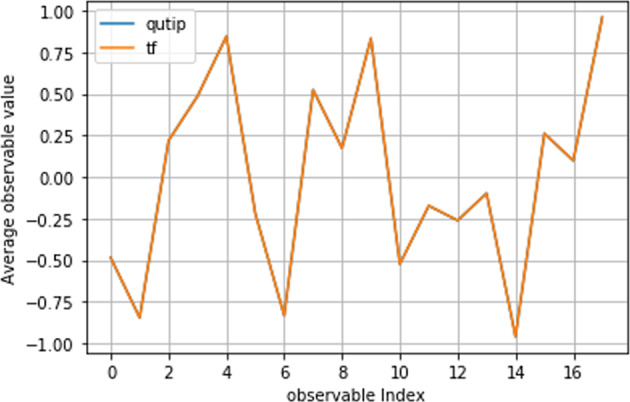
Plot of average observable (measurement) value for all observables (index indicates each observable in order of Pauli measurements) for all measurement outcomes for samples drawn from dataset G_1q_X (using TensorFlow ‘tf’, orange line) against the same mean for equivalent simulations in Qutip (blue line - not shown due to identical overlap) for a single dataset. Each dataset was sampled and comparison against Qutip was undertaken with equivalent results. The error between means was of order 10^−6^ i.e. they were effectively identical (so the blue line is not shown).

**Fig. 4:**
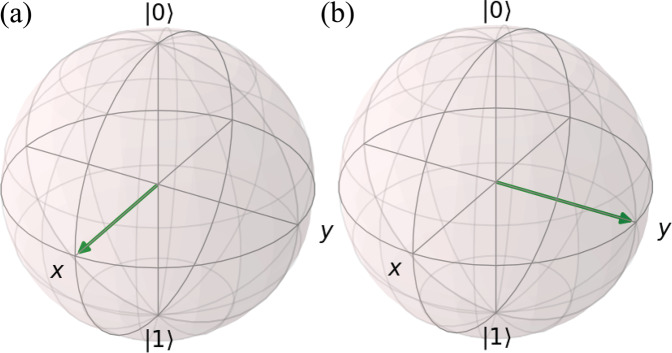
An example of a quantum state rotation on the Bloch sphere. The |0 > 0, |1⟩ indicates the *σ*_*z*_-axis, the *X* and *Y* the *σ*_*x*_ and *σ*_*y*_ axes respectively. In (a), the vector is residing in a +1 *σ*_*x*_ eigenstate. By rotating about the *σ*_*z*_ axis by π/4, the vector is rotated to the right, to the +1 *σ*_*y*_ eigenstate. A rotation about the *σ*_*Z*_ axis by angle *θ* is equivalent to the application of the unitary $U(\theta )=\exp (-i{\theta }_{z}{\sigma }_{z}/2)$.

The second and primary technical validation of the QDataSet was undertaken by comparing mean expectation values of observables for subsamples of each of the datasets against the equivalent expectations for simulations and measurements undertaken in using Qutip^34^. To generate the Qutip equivalents, the equivalent parameters (e.g. Hamiltonian parameters, pulse parameters) were input into Qutip to generate the relevant outputs. For each dataset in the QDataSet, the verification procedure was run on varying samples. To undertake this process, we adopted two validation strategies Figs. 3, 4.

- *Mean expectation of all observables over all noise realisations*. In this case, for a sample of examples from each dataset in the QDataSet, the mean expectation over all noise realisations for all observables (i.e. measurements) was compared against the same mean measurements for the equivalent simulation generated in Qutip. This was done for the noiseless and noisy case. The two means were then compared. On average the error (difference between the means) of the order 10^−06^, demonstrating the equivalence of the QDataSet simulation code with that from Qutip. A plot is set out in  Fig. 3.
- *Mean expectation of single observables over separate noise realisations*. In the second case, the mean expectation over all noise realisations for each separate observable was compared against the same mean measurements for the equivalent simulation generated in Qutip. Again, this was done for the noiseless and noisy case. Comparison of the two means showed that on average the error (difference between the means) of the order 10^−07^, in turn demonstrating the equivalence of the QDataSet simulation code with that from Qutip.

## Usage Notes

### Overview

In this section, we include further usage notes related to the comprises 52 QDataSet datasets based on simulations of one- and two-qubit systems evolving in the presence and/or absence of noise subject to a variety of controls. The QDataSet has been developed primarily for use in training, benchmarking and competitive development of classical and quantum algorithms for common tasks in quantum control, quantum tomography and noise spectroscopy. It has been generated using customised code drawing upon base-level Python packages in order to facilitate interoperability and portability across common machine learning and quantum programming platforms. Each dataset consists of 10,000 samples which in turn comprise a range of data relevant to the training of machine learning algorithms for solving optimisation problems. The data includes a range of information (stored in list, matrix or tensor format) regarding quantum systems and their evolution, such as: quantum state vectors, drift and control Hamiltonians and unitaries, Pauli measurement distributions, time series data, pulse sequence data for square and Gaussian pulses and noise and distortion data. The total compressed size of the QDataSet (using Pickle and zip formats) is around 14TB (uncompressed, well-over 100TB). Researchers can use the QDataSet in a variety of ways to design algorithms for solving problems in quantum control, quantum tomography and quantum circuit synthesis, together with algorithms focused on classifying or simulating such data. We also provide working examples of how to use the QDataSet in practice and its use in benchmarking certain algorithms. Each part below provides in-depth detail on the QDataSet for researchers who may be unfamiliar with quantum computing, together with specifications for domain experts within quantum engineering, quantum computation and quantum machine learning.

The aim of generating the datasets is threefold: (a) simulating typical quantum engineering systems, dynamics and controls used in laboratories; (b) using such datasets as a basis to train machine learning algorithms to solve certain problems or achieve certain objectives, such as attainment of a quantum state *ρ*, quantum circuit *U* or quantum control problem generally (among others); and (c) enable optimisation of algorithms and spur development of optimised algorithms for solving problems in quantum information, analogously with the role of large datasets in the classical setting. We explain these use cases in more detail below:

1. *Datasets as simulations*. Firstly, we have aimed to generate datasets which abstractly simulate the types of data, characteristics and features which would be commonly used in laboratories and experimental setups. Each dataset is an abstractions (say of particular Hamiltonians, or noise profiles) which can have any number of physical *realisations* depending on the experimental design. So different experiments can ultimately realise, in the abstract the same or a sufficiently similar structure as that provided by the data. This is an important design choice relating to how the QDataSet is intended to be used. For example, the implementation of the particular Hamiltonians or state preparation may be done using trapped-ion setups, quantum dot or transmon-based qubits^35^, doped systems or otherwise. We assume the availability of a mapping between the dataset features, such as the controls pulses, and particular control devices (such as voltage or microwave-based controls), for example, in the laboratory.
2. *Training algorithms using datasets*. The second usage case for the QDataSet is related but distinct from the first. The aim is that training models using the datasets has applicability to experimental setups. Thus, for example, a machine learning model trained using the datasets in theory should provide, for example, the optimal set of pulses or interventions needed to solve (and, indeed, optimise) for some objective. It is intended that the output of the machine learning model is an abstraction which can then be realised via the specific experimental setup. The aim then is that the abstraction of each experiments setup allows the application of a variety of machine learning models for optimising in a way that is directly applicable to experimental setups, rather than relying upon experimentalists to then work-out how to translate the model’s output into their particular experimental context. Requiring conformity of outputs within these abstract criteria thus facilitates a greater, practical, synthesis between machine learning and the implementation of solutions and procedures in experiments.
3. *Benchmarking, development and testing*. The third primary use of the datasets is to provide a basis for benchmarking, development and testing of existing and new algorithms in quantum machine learning for quantum control, tomography and related to noise mitigation. As discussed above, classical machine learning has historically been characterised by the availability of large-scale datasets with which to train and develop algorithms. The role of these large datasets is multifaceted: (i) they provide a means of *benchmarking* algorithms (see above), such that a common set of problem parameters, constraints and objectives allows comparison among different models; (ii) their size often means they provide a richer source of overt and latent (or constructible) features which machine learning models may draw upon, improving the versatility and diversity of models which may be usefully trained. The aim of the QDataSet is then that it can be used in tandem by researchers as benchmarking tool for algorithms which they may wish to apply to their own data or experiments.

### Machine learning using the QDataSet

There are many problems related to the characterization and control of quantum systems that can be solved using machine learning techniques. In this section, try to give an overview on a number of such problems and how to approach them using machine learning. Here we provide a brief overview of the different types of problems in quantum computing, engineered quantum systems and quantum control for which the QDataSet and algorithms trained using it may be useful. The list is necessarily non-exhaustive and is intended to provide some direction mainly to machine learning researchers unfamiliar with key problems in applied quantum science.

### Benchmarking

Benchmarking algorithms using standardised datasets is an important developmental characteristics of classical machine learning. Benchmarks provide standardised datasets, preprocessing protocols, metrics, architectural features (such as optimisers, loss functions and regularisation techniques) which ultimately enable research communities to precisify their research contributions and improve upon state of the art results. Results in classical machine learning are typically presented by comparison with known benchmarks in the field and adjudged by the extent to which they outperform the current state of the art benchmarks. Results are presented in tabular format with standardised metrics for comparison, such as accuracy, F1-score or AUC/ROCR statistics. The QDataSet has been designed with these metrics in mind. For example, a range of classical or quantum statistics (e.g. fidelity) can be used to benchmark the performance of algorithms that use the datasets in training. The role of benchmarking is important in classical contexts. Firstly, it enables a basis for researchers across machine learning subdisciplines to gauge the extent to which their results correlate to algorithmic design as distinct from unique features of training data or use cases. Secondly, it provides a basis for better assessing the algorithmic state of the art within subfields. Given its relative nascency, QML literature tends to focus on providing proof-of-concept examples as to how classical, hybrid or quantum-native algorithms can be used for classification or regression tasks. There is little in the way of systematic benchmarking of QML algorithms against their classical counterparts in terms of performance of specifically machine learning algorithms.

Recent examples in a QML setting of benchmarking include comparisons of using different error distributions relevant to quantum chemistry (and how these affect performance)^36^, benchmarking machine learning algorithms for adaptive phase estimation^37^ and generative machine learning with tensor networks^38^. In quantum information science more broadly, comparison with classical algorithms is often driven from computational complexity considerations and the search for quantum supremacy or outperformance, namely whether there exists a classical algorithm which can achieve results with equivalent efficiency of the quantum algorithm. Users of the QDataSet for QML research into quantum control, tomography or noise mitigation would benefit from adopting (and adapting) practices common in classical machine learning when reporting results, especially the inclusion of benchmarks against leading state of the art algorithms for particular use-cases, such as classification or regression tasks. Selecting the appropriate benchmarking algorithms itself tends to benefit from domain expertise. The QDataSet has been designed in order to be benchmarked against both classical and quantum algorithms.

#### Benchmarking by learning protocol

Typically machine learning algorithm classification is based firstly on whether the learning protocols are *supervised*, *unsupervised* or semi-supervised^25,39^. *Supervised learning* uses known input and output (label) data to train algorithms to estimate label data. Algorithmic models are updated according to an optimisation protocol, typically gradient descent, in order to achieve some objective, such as minimisation of a loss function that compares the similarity of estimates to label data. *Unsupervised learning*, by contrast, is a learning protocol where label or classification of data is unknown and must be estimated via grouping or clustering together in order to ascertain identifying features. Common techniques include clustering, dimensionality reduction techniques or graph-based methods. *Semi-supervised* learning is an intermediate algorithmic classification drawing on aspects of both supervised and unsupervised learning protocols. Usually known label data, say where only part of a dataset is labelled or classified, is included in architectures in order to learn the classifications which in turn can be used in a supervised contexts. The QDataSet can be used in a variety of supervised, unsupervised or semi-supervised contexts. For example, training an algorithm for optimal quantum control can be undertaken in a supervised context (using pulse data, measurement statistics or Hamiltonian sequences) as label data and modelling estimates accordingly. Alternatively, semi-supervised or unsupervised protocols for tomographic classification can be trained using the QDataSet. In any case, an understanding of standard and state of the art algorithms in each category can provide QML researchers using the QDataSet with a basis for benchmarking their own algorithms and inform the design of especially hybrid approaches (see ref. ^40^. for an overview and for quantum examples of the above).

#### Benchmarking by objectives and architecture

The choice of benchmarking algorithms will also be informed by the objectives and architecture. Classically, algorithms can be parsed into various categories. Typically they are either *regression*-based algorithms, used where the objective is to estimate (and minimise error in relation to) continuous data or *classification*-based algorithms, where the objective is to classify data into discrete categories. *Regression algorithms* are algorithms that seek to model relationships between input variables and outputs iteratively by updating models (and estimates) in order to minimise error between estimates and label data. Typical regression algorithms usually fall within broader families of generalised linear models (GLMs)^41^ and include algorithms such as ordinary least squares, linear and logistic regression, logit and probit models, multivariate models and other models depending on link functions of interest. GLMs are also characterised by regularisation techniques that seek to optimise via penalising higher complexity, outlier weights or high variance. GLMs offer more flexibility for use in QML and for using the QDataSet in particular as they are not confined to assuming errors are normally distributed. Other approaches using Bayesian methods, such as naive Bayes, Gaussian Bayes, Bayesian networks and averaged one-dependence estimators provide yet further avenues for benchmarking algorithms trained on the QDataSet for classification or regression tasks. *Classification* models aim to solve decision problems via classification. They typically compare new data to existing datasets using a metric or distance measure. Examples include clustering algorithms such as k-nearest neighbour, support vector machines, learning vector quantisation, decision-trees, locally weighted learning, or graphical models using spatial filtering. Most of the algorithms mentioned thus far fall within traditional machine learning.

Over the last several decades or so, *neural network* architectures have emerged as a driving force of machine learning globally. Quantum analogues and hybrid neural network architecture has itself a relatively long lineage, including quantum analogues of perceptrons, quantum neural networks, quantum hopfield networks (see refs. ^40,42^) through to modern deep learning architectures (such as convolutional, recurrent, graphical and hierarchical neural networks and generative models^39^). One feature of algorithmic development that is particularly important is dealing with the curse of dimensionality - and in a quantum context, barren plateaux. Common techniques to address such problems include dimensionality reduction techniques or symmetry-based (for example, tensor network) techniques whose ultimate goal is to reduce datasets down to their most informative structures while maintaining computational feasibility. While the QDataSet only extends to two-qubit simulations, the size and complexity of the data suggests the utility of dimensionality-reduction techniques for particular problems, such as tomographic state characterisation. To this end, algorithms developed using the QDataSet can benefit from benchmarking and adapting classical dimensionality-reduction techniques, such as principal component analysis, partial regression, singular value decompositions, matrix factorisation and other techniques^25^. It is also important to mention that there has been considerable work in QML generally toward the development of quantum and hybrid analogues of such techniques. These too should be considered when seeking benchmarks.

Finally, it is worth mentioning the use (and importance) of ensemble methods in classical machine learning. Ensemble methods tend to combine what are known as ‘weak learner’ algorithms into an ensemble which, in aggregate, outperforms any individual instance of the algorithm. Each weak learner’s performance is updated by reference to a subset of the population of weak learners. Such techniques would be suitable for use when training algorithms on the QDataSet. Popular examples of such algorithms are gradient-boosting algorithms, such as xGboost^43^.

### Example applications of the QDataSet

In this section, we outline a number of applications for which the QDataSet can be used. These include training machine learning algorithms for use in quantum state (or process) tomography, quantum noise spectroscopy and quantum control. The QDataSet repository contains a number of example Jupyter notebooks corresponding to the examples below. The idea behind these datasets is that machine learning practitioners can input their own algorithms into the code to run experiments and test how well their algorithms perform.

#### Quantum state tomography

Quantum state tomography involves reconstructing an estimate $\widehat{\rho }$ of the state of a quantum system given a set of measured observables. The quantum state of interest may be in either a mixed or pure state and the task is to uniquely identify the state among a range of potential states. Tomography requires that measurements be *tomographically complete*, which means that the set of measurement operators form a basis for the Hilbert space of interest. Abstractly, the problem involves stipulating a set of operators {*O*_*i*_}_*i*_ as input, and the corresponding desired target outputs {〈*O*_*i*〉_}_*i*_. The objective is to find the best model that fits this data. We know that the relation between these is given by 〈*O*_*i*_〉 = Tr(*ρO*_*i*_) and we can use this fact to find the estimate of the state. Tomography requires repeatedly undertaking different measurements upon quantum states described by identical density matrices which in turn gives rise to a measurement distribution from which probabilities of observables can be inferred. Such inferred probabilities are used to in turn construct a density matrix consistent with observed measurement distributions thus characterising the state. More formally, assuming an informationally complete positive-operator valued measure (POVM - see Supplementary Information for theoretical background) {*O*_*i*_} spanning the Hilbert-Schmidt space $B({\mathscr{H}})$ of operators on ${\mathscr{H}}$, we can write the probability of an observation *i* given density matrix *ρ* using the Hilbert-Schmidt norm above i.e:

31 $$p(i| \rho )={O}_{i}={\rm{Tr}}(\rho {O}_{i})$$

Data are gathered from a discrete set of *M* experiments, where each experiment is a process of initial state preparation, by applying a sequence of gates {*G*_*j*_} and measuring. This experimental process and measurement is repeated *N* times leading to a frequency count *n*_*i*_ of a particular observable *i*. The probability of that observable is then estimated as:

$$p(i| \rho )\approx \frac{{n}_{i}}{N}={\widehat{p}}_{i}$$

from which we reconstruct the density matrix *ρ*. Quantum process tomography is a related but distinct type of tomography. In this case, we also have a set of test states {*ρ*_*j*_} which span $B({\mathscr{H}})$. To undertake process tomography, an unknown gate sequence *G*_*k*_ comprising *K* gates is applied to the states such that:

32 $$p(i| G,{\rho }_{j})\approx \frac{{n}_{i}}{N}={\widehat{p}}_{j,i}$$

The QDataSet can be used to train algorithms for machine learning algorithms for tomography. Quantum state and process tomography is particularly challenging. One must ensure that the estimate we get is physical, i.e. positive semidefinite with unit trace. Furthermore, the number of measurements *N* required for sufficient precision to completely characterise *ρ* scales rapidly. Each of the *K* gates in a sequence *G*_*k*_ requires *d*^2^(*d* − 1) (where $d={\rm{\dim }}| B({\mathscr{H}})|$) experiments (measurements) to sufficiently characterise the quantum process is *Kd*^4^ − (*K* − 2)*d*^2^ − 1 (see ref. ^44^ for more detail). Beyond a small number of qubits, it becomes computationally infeasible to completely characterise states by direct measurement, thus parametrised or incomplete tomography must be relied upon. Machine learning techniques naturally offer potential to assist with such optimisation problems in tomography, especially neural network approaches where inherent non-linearities may enable sufficient approximations that traditional tomographic techniques may not. Examples of the use of classical machine learning include demonstration of improvements due to neural network-based (non-linear) classifiers over linear classifiers for tomography tasks^45^ and classical convolutional neural networks to assess whether a set of measurements is informationally complete^46^.

The objective of an algorithm trained using the QDataSet may be, for example, be to predict (within tolerances determined by the use case) the tomographic description of a final quantum state from a limited set of measurement statistics (to avoid having to undertake *N* such experiments for large *N*). Each of the one- and two-qubit datasets is informationally complete with respect to the Pauli operators (and identity) i.e. can be decomposed into a one- and two-dimensional Pauli basis. There are a variety of objectives and techniques which may be adopted. Each of the 10,000 examples for each profile constitutes an experiment comprising initial state preparation, state evolution and measurement. One approach using the QDataSet would be to try to produce an estimate $\widehat{\rho }(T)$ of the final state *ρ*(*T*) (which can be reconstructed by application of the unitaries in the QDataSet to the initial states) using the set of Pauli measurements {*E*_*O*_}. To train an algorithm for tomography without a full set of *N* measurements being undertaken, on can stipulate the aim of the machine learning algorithm is then to take a subset of those Pauli measurements as input and try to generate a final state $\widehat{\rho }(T)$ that as closely approximates the known final state *ρ*(*T*) provided by the QDataSet.

A variety of techniques can be used to draw from the measurement distributions and iteratively update the estimate $\widehat{\rho }(T)$, for example gradient-based updating of such estimates^47^. The distance measure could be any number of the quantum metrics described in the Supplementary Information, including state or operator fidelity, trace distance of quantum relative entropy. Classical loss functions, such as MSE or RMSE can then be used (as is familiar to machine learning practitioners) to construct an appropriate loss function for minimisation. A related, but alternative, approach is to use batch fidelity where the loss function is to minimise the error between a vector of ones and fidelities, the vector being the size of the relevant batch. Similar techniques may also be used to develop tools for use in gate set tomography, where the sequence of gates *G*_*k*_ is given by the sequence of unitaries *U*_0_ in the QDataSet. In that case, the objective would be to train algorithms to estimate *G*_*k*_ given the set of measurements, either in the presence of absence of noise. Table (2) sets out an example summary for using the QDataSet for tomography.

**Table 2:** QDataSet features for quantum state tomography.

Item	Description
Objective	Algorithm to learn characterisation of state *ρ* given measurements {*E*_*O*_}.
Inputs	Set of Pauli measurements {*E*_*O*_}, one for each of the *M* experiments (in the QDataSet, this is
Label	Final state *ρ*(*T*)
Intermediate inputs	Hamiltonians, Unitary operators, Initial states *ρ*_0_
Output	Estimate of final state $\widehat{\rho }(T)$
Metric	State fidelity $F(\rho ,\widehat{\rho })$, Quantum relative entropy

The left columns lists typical categories in a machine learning architecture. The right column describes the corresponding feature(s) of the QDataSet that would fall into such categories for the use of the QDataSet in training quantum tomography algorithms.

#### Quantum noise spectroscopy

The QDataSet can be used to develop and test machine algorithms to assist with noise spectroscopy. In this problem, we are interested in finding models of the noise affecting a quantum system given experimental measurements. In terms of the *V*_*O*_ operators discussed earlier, we would like to find an estimate of *V*_*O*_ given a set of control pulse sequences, and the corresponding observables. The QDataSet provides a sequence of *V*_*O*_ operators encoding the average effect of noise on measurement operators. This set of data can be used to train algorithms to estimate *V*_*O*_ from noisy quantum data, such as noisy measurements or Hamiltonians that include noise terms. An example approach includes as follows and proceeds from the principle that we have known information about quantum systems that can be input into the algorithmic architecture (initial states, controls, even measurements) and we are trying to estimate unknown quantities (the noise profile). The inputs to the algorithm would include: the initial quantum states, in the QDataSet case the initial states (being eigenstates of the Pauli operators). Intermediate inputs would include the system and noise Hamiltonians *H*_0_,*H*_1_ and/or the system and noise unitaries *U*_0_,*U*_1_. Alternatively, inputs could also include details of the various noise realisations. The type of inputs will depend on the type of applied use case, such as how much information may be known about noise sources. Label data would be the set of measurements {*E*_*O*_} (expectations of the observables). Given the inputs (control pulses) and outputs, the problem becomes estimating the mapping {*V*_*O*_}, such that inputs are mapped to outputs via Eq. (13). Note that details about noise realisations or distributions are never accessible experimentally.

Alternatively, architectures may take known information about the system such as Pauli measurements as inputs. Another approach is to adopt a similar architecture to that in refs. ^13,47^. and construct a multi-layered architecture that replicates the simulation, where the $\{{\widehat{V}}_{O}\}$ are extracted from intermediate or custom layers in the architecture. Such greybox approaches may combine traditional or deep-learning methods and have the benefit of providing finer-grained control over algorithmic structure by allowing, for example, the encoding of ‘whitebox’ or known processes from quantum physics (thereby eliminating the need for the algorithm to learn these processes). Table (3) sets out one example approach that may be adopted.

**Table 3:** QDataSet features for quantum noise spectroscopy.

Item	Description
Objective	Algorithm to estimate noise operators $\{{V}_{O}\}$, thereby characterising relevant features of noise affecting quantum system.
Inputs	Pulse sequence, reconstructed from the *pulse_parameters* feature in the dataset.
Label	Set of measurements $\{{E}_{O}\}$
Intermediate inputs	Hamiltonians, Unitary operators, Initial states ${\rho }_{0}$
Output	Estimate of measurements $\left\{{\widehat{E}}_{O}\right\}$
Metric	MSE (between estimates and label data)
	$$MSE\left({E}_{O},{\widehat{E}}_{O}\right)$$ (34)

The left columns lists typical categories in a machine learning architecture. The right column describes the corresponding feature(s) of the QDataSet that would fall into such categories for the use of the QDataSet in training quantum tomography algorithms.

#### Quantum control and circuit synthesis

The QDataSet has been designed in particular to facilitate algorithmic design for quantum control. As described in some detail above, we wish to compare different (hybrid and classical) machine learning algorithms to optimise a typical problem in quantum control, namely describing the optimal sequence of pulses in order to synthesise a target unitary *U*_*T*_ of interest. Here the datasets form the basis of training, validation and test sets used to train and verify each algorithm. The target (label) data for quantum control problems can vary. Typically, the objective of quantum control is to achieve a reachable state *ρ*(*T*) via the application of control functions to generators, such as Pauli operators. Achieving the objective means developing an algorithm that outputs a sequence of control functions which in turn describe the sequence of experimental controls *f*_*α*_(*t*). A typical machine learning approach to quantum control takes *ρ*(*T*) as an input together with intermediate inputs, such as the applicable generators (e.g. Pauli operators encoded in the system Hamiltonian *H*_0_(*t*) of the QDataSet). The algorithm must learn the appropriate time-series distribution of *f*_*α*_(*t*) (the set of control pulses included in the QDataSet, their amplitude and sequence in which they should be applied) in order to synthesise the estimate $\widehat{\rho }(T)$. Some quantum control problems are agnostic as to the quantum circuit pathway (sequence of unitaries) taken to reach $\widehat{\rho }(T)$, though usually the requirement is that the circuit be resource optimal in some sense, such as time-optimal (shortest time) or energy-optimal (least energy).

One approach is to treat *f*_*α*_(*t*) as the label data and *ρ*(*T*) as input data to try to learn a mapping between them. A naive blackbox approach is unlikely to efficiently solve this problem as it would require learning from scratch solutions to the Schrödinger equation. A more efficient approach may be to encode known information, such as the laws governing Hamiltonian evolution etc into machine learning architecture, such as greybox approaches described above. In this case, the target *f*_*α*_(*t*) must be included as an intermediate input into the system Hamiltonians governing the evolution of *ρ*(*T*), yet remains the output of interest. In such approaches, the input data would be the initial states of the QDataSet with the label data being *ρ*(*T*) (and label estimate $\widehat{\rho }(T)$). Applicable loss functions then seek to minimise the (metric) distance between *ρ*(*T*) and $\widehat{\rho }(T)$, such as fidelity $F(\rho (T),\widehat{\rho }(T))$. To recover the sought after sequence *f*_*α*_(*t*), the architecture then requires a way to access the intermediate state of parameters representing *f*_*α*_(*t*) within the machine learning architecture.

If path specificity is not important for a use case, then trained algorithms may synthesise any pathway to achieve $\widehat{\rho }(T)$, subject to the optimisation constraints. The trained algorithm need not replicate the pathways taken to reach *ρ*(*T*) in the training data. If path specificity is desirable, then the QDataSet intermediate operators *U*_0_(*t*) and *U*_1_(*t*) can be used to reconstruct the intermediate states i.e. to recover the time-independent approximation:

33 $$U{(t)}^{\dagger }\rho U(t)\approx ({U}_{n}\ldots {U}_{1})\rho ({U}_{1}\ldots {U}_{n})$$

An example of such an approach is in ref. ^14^. where time-optimal quantum circuit data, representing geodesics on Lie group manifolds, is used to train algorithms for generating time-optimal circuits. Table (4) sets out schemata for using the QDataSet in a quantum control context.

**Table 4:** QDataSet features for quantum control.

Item	Description
Objective	Algorithm to learn optimal sequence of controls to reach final state $\rho (T)$ or (equivalently) synthesise target unitary ${U}_{T}$.
Inputs	Hamiltonians containing Pauli generators ${H}_{0}(t)$
Label	Final state $\rho (T)$ and (possibly) intermediate states $\rho ({t}_{j})$ for each time-interval ${t}_{j}$.
Intermediate fixed inputs	Sequence of unitary operators ${U}_{0}(t),{U}_{1}(t)$, Initial states ${\rho }_{0}$
Intermediate weights	Sequence of pulses ${f}_{\alpha }(t)$ including parameters depending on whether square or Gaussian (for example)
Output	Estimate of final state $\widehat{\rho }(T)$ and intermediate states $\widehat{\rho }({t}_{j})$
Metric	Average operator fidelity $F(\rho ,\widehat{\rho })$

The left columns lists typical categories in a machine learning architecture. The right column describes the corresponding feature(s) of the QDataSet that would fall into such categories for the use of the QDataSet in training quantum control algorithms. The specifications are just one of a set of possible ways of framing quantum control problems using machine learning.

**Table 5:** The general categorization of the provided datasets.

Category	Qubits	Drift	Control	Noise
1	1	(*z*)	(*x*)	(*z*)
2	1	(*z*)	(*x*, *y*)	(*x*, *z*)
3	2	(*z*1,1*z*)	(*x*1,1*x*)	(*z*1,1*z*)
4	2	(*z*1,1*z*)	(*x*1,1*x*,*xx*)	(*z*1,1*z*)

The QDataSet examples were generated from simulations of either one or two qubit systems. For each one or two qubit simulation, the drift component of the Hamiltonian was along a particular axis (the *z*-axis) for the single-qubit case and the *z*-axis of the first qubit for the two-qubit case (but not the second qubit) or vice versa. Controls were applied along different axes, such as *x*- or *y*- axes. Finally, noise was similarly added to different axes: the *z*-axis (and in some cases the *x*-axis) of the single qubit case and the *z*-axis case of the first or second qubit for the two-qubit case.

**Table 6:** Dataset Parameters: *T*: total time, set to unity for standardisation; *M*: the number of time-steps (discretisations); *K*: the number of noise realisations; Ω: the energy gap for the single qubit case (where subscripts 1 and 2 represent the energy gap for each qubit in the single qubit case); *n*: number of control pulses; *A*_max_, *A*_min_: maximum and minimum amplitude; *σ*: standard deviation of pulse spacing (for Gaussian pulses).

Parameter	Value
*T*	1
*M*	1024
*K*	2000
Ω	12
Ω_1_	12
Ω_2_	10
*n*	5
*A*_min_	−100
*A*_max_	100
*σ*	T/(12 M)

**Table 7:** QDataSet File Description (Gaussian). The left column identifies each dataset in the respective QDataSet examples while the description column describes the profile of the Gaussian pulse datasets in terms of (i) number of qubits, (ii) axis of control and pulse wave-form (iii) axis and type of noise and (iv) whether distortion is present or absent.

Dataset	Description
G_1q_X	(i) Qubits: one; (ii) Control: *x*-axis, Gaussian; (iii) Noise: none; (iv) No distortion.
G_1q_X_D	(i) Qubits: one; (ii) Control: *x*-axis, Gaussian; (iii) Noise: none; (iv) Distortion.
G_1q_XY	(i) Qubits: one; (ii) Control: *x*-axis and *y*-axis, Gaussian; (iii) Noise: none; (iv) No distortion.
G_1q_XY_D	(i) Qubits: one; (ii) Control: *x*-axis and *y*-axis, Gaussian; (iii) Noise: none; (iv) Distortion.
G_1q_XY_XZ_N1N5	(i) Qubits: one; (ii) Control: *x*-axis and *y*-axis, Gaussian; (iii) Noise: N1 on *x*-axis, N5 on *z*-axis; (iv) No distortion.
G_1q_XY_XZ_N1N5_D	(i) Qubits: one; (ii) Control: *x*-axis and *y*-axis, Gaussian; (iii) Noise: N1 on *x*-axis, N5 on *z*-axis; (iv) No distortion.
G_1q_XY_XZ_N1N6	(i) Qubits: one; (ii) Control: *x*-axis and *y*-axis, Gaussian; (iii) Noise: N1 on *x*-axis, N6 on *z*-axis; (iv) Distortion.
G_1q_XY_XZ_N1N6_D	(i) Qubits: one; (ii) Control: *x*-axis and *y*-axis, Gaussian; (iii) Noise: N1 on *x*-axis, N6 on *z*-axis; (iv) No distortion.
G_1q_XY_XZ_N3N6	(i) Qubits: one; (ii) Control: *x*-axis and *y*-axis, Gaussian; (iii) Noise: N1 on *x*-axis, N6 on *z*-axis; (iv) Distortion.
G_1q_XY_XZ_N3N6_D	(i) Qubits: one; (ii) Control: *x*-axis and *y*-axis, Gaussian; (iii) Noise: N1 on *x*-axis, N6 on *z*-axis; (iv) No distortion.
G_1q_X_Z_N1	(i) Qubits: one; (ii) Control: *x*-axis, Gaussian; (iii) Noise: N1 on *z*-axis; (iv) No distortion.
G_1q_X_Z_N1_D	(i) Qubits: one; (ii) Control: *x*-axis, Gaussian; (iii) Noise: N1 on *z*-axis; (iv) Distortion.
G_1q_X_Z_N2	(i) Qubits: one; (ii) Control: *x*-axis, Gaussian; (iii) Noise: N2 on *z*-axis; (iv) No distortion.
G_1q_X_Z_N2_D	(i) Qubits: one; (ii) Control: *x*-axis, Gaussian; (iii) Noise: N2 on *z*-axis; (iv) Distortion.
G_1q_X_Z_N3	(i) Qubits: one; (ii) Control: *x*-axis, Gaussian; (iii) Noise: N3 on *z*-axis; (iv) No distortion.
G_1q_X_Z_N3_D	(i) Qubits: one; (ii) Control: *x*-axis, Gaussian; (iii) Noise: N3 on *z*-axis; (iv) Distortion.
G_1q_X_Z_N4	(i) Qubits: one; (ii) Control: *x*-axis, Gaussian; (iii) Noise: N4 on *z*-axis; (iv) No distortion.
G_1q_X_Z_N4_D	(i) Qubits: one; (ii) Control: *x*-axis, Gaussian; (iii) Noise: N4 on *z*-axis; (iv) Distortion.
G_2q_IX-XI_IZ-ZI_N1-N6	(i) Qubits: two; (ii) Control: *x*-axis on both qubits, Gaussian; (iii) Noise: N1 and N6 *z*-axis on each qubit; (iv) No distortion.
G_2q_IX-XI_IZ-ZI_N1-N6_D	(i) Qubits: two; (ii) Control: *x*-axis on both qubits, Gaussian; (iii) Noise: N1 and N6 *z*-axis on each qubit; (iv) Distortion.
G_2q_IX-XI-XX	(i) Qubits: two; (ii) Control: single *x*-axis control on both qubits and *x*-axis interacting control, Gaussian; (iii) Noise: none; (iv) No distortion.
G_2q_IX-XI-XX_D	(i) Qubits: two; (ii) Control: single *x*-axis control on both qubits and *x*-axis interacting control, Gaussian; (iii) Noise: none; (iv) Distortion.
G_2q_IX-XI-XX_IZ-ZI_N1-N5	(i) Qubits: two; (ii) Control: single *x*-axis control on both qubits and *x*-axis interacting control, Gaussian; (iii) Noise: N1 and N5 on *z*-axis noise on each qubit; (iv) No distortion.
G_2q_IX-XI-XX_IZ-ZI_N1-N5	(i) Qubits: two; (ii) Control: single *x*-axis control on both qubits and *x*-axis interacting control, Gaussian; (iii) Noise: N1 and N5 on *z*-axis noise on each qubit; (iv) Distortion.

**Table 8:** QDataSet File Description (Square).

Dataset	Description
S_1q_X	(i) Qubits: one; (ii) Control: *x*-axis, square; (iii) Noise: none; (iv) No distortion.
S_1q_X_D	(i) Qubits: one; (ii) Control: *x*-axis, square; (iii) Noise: none; (iv) Distortion.
S_1q_XY	(i) Qubits: one; (ii) Control: *x*-axis and *y*-axis, square; (iii) Noise: none; (iv) No distortion.
S_1q_XY_D	(i) Qubits: one; (ii) Control: *x*-axis and *y*-axis, square; (iii) Noise: none; (iv) Distortion.
S_1q_XY_XZ_N1N5	(i) Qubits: one; (ii) Control: *x*-axis and *y*-axis, square; (iii) Noise: N1 on *x*-axis, N5 on *z*-axis; (iv) No distortion.
S_1q_XY_XZ_N1N5_D	(i) Qubits: one; (ii) Control: *x*-axis and *y*-axis, Gaussian; (iii) Noise: N1 on *x*-axis, N5 on *z*-axis; (iv) No distortion.
S_1q_XY_XZ_N1N6	(i) Qubits: one; (ii) Control: *x*-axis and *y*-axis, square; (iii) Noise: N1 on *x*-axis, N6 on *z*-axis; (iv) Distortion.
S_1q_XY_XZ_N1N6_D	(i) Qubits: one; (ii) Control: *x*-axis and *y*-axis, square; (iii) Noise: N1 on *x*-axis, N6 on *z*-axis; (iv) No distortion.
S_1q_XY_XZ_N3N6	(i) Qubits: one; (ii) Control: *x*-axis and *y*-axis, square; (iii) Noise: N1 on *x*-axis, N6 on *z*-axis; (iv) Distortion.
S_1q_XY_XZ_N3N6_D	(i) Qubits: one; (ii) Control: *x*-axis and *y*-axis, square; (iii) Noise: N1 on *x*-axis, N6 on *z*-axis; (iv) No distortion.
S_1q_X_Z_N1	(i) Qubits: one; (ii) Control: *x*-axis, square; (iii) Noise: N1 on *z*-axis; (iv) No distortion.
S_1q_X_Z_N1_D	(i) Qubits: one; (ii) Control: *x*-axis, square; (iii) Noise: N1 on *z*-axis; (iv) Distortion.
S_1q_X_Z_N2	(i) Qubits: one; (ii) Control: *x*-axis, square; (iii) Noise: N2 on *z*-axis; (iv) No distortion.
S_1q_X_Z_N2_D	(i) Qubits: one; (ii) Control: *x*-axis, Gaussian; (iii) Noise: N2 on *z*-axis; (iv) Distortion.
S_1q_X_Z_N3	(i) Qubits: one; (ii) Control: *x*-axis, square; (iii) Noise: N3 on *z*-axis; (iv) No distortion.
S_1q_X_Z_N3_D	(i) Qubits: one; (ii) Control: *x*-axis, square; (iii) Noise: N3 on *z*-axis; (iv) Distortion.
S_1q_X_Z_N4	(i) Qubits: one; (ii) Control: *x*-axis, square; (iii) Noise: N4 on *z*-axis; (iv) No distortion.
S_1q_X_Z_N4_D	(i) Qubits: one; (ii) Control: *x*-axis, square; (iii) Noise: N4 on *z*-axis; (iv) Distortion.
S_2q_IX-XI_IZ-ZI_N1-N6	(i) Qubits: two; (ii) Control: *x*-axis on both qubits, square; (iii) Noise: N1 and N6 *z*-axis on each qubit; (iv) No distortion.
S_2q_IX-XI_IZ-ZI_N1-N6_D	(i) Qubits: two; (ii) Control: *x*-axis on both qubits, square; (iii) Noise: N1 and N6 *z*-axis on each qubit; (iv) Distortion.
S_2q_IX-XI-XX	(i) Qubits: two; (ii) Control: single *x*-axis control on both qubits and *x*-axis interacting control, square; (iii) Noise: none; (iv) No distortion.
S_2q_IX-XI-XX_D	(i) Qubits: two; (ii) Control: single *x*-axis control on both qubits and *x*-axis interacting control, square; (iii) Noise: none; (iv) Distortion.
S_2q_IX-XI-XX_IZ-ZI_N1-N5	(i) Qubits: two; (ii) Control: *x*-axis on both qubits and *x*-axis interacting control, square; (iii) Noise: N1 and N5 *z*-axis on each qubit; (iv) No distortion.
S_2q_IX-XI-XX_IZ-ZI_N1-N5_D	(i) Qubits: two; (ii) Control: *x*-axis on both qubits and *x*-axis interacting control, square; (iii) Noise: N1 and N5 *z*-axis on each qubit; (iv) Distortion.
S_2q_IX-XI-XX_IZ-ZI_N1-N6	(i) Qubits: two; (ii) Control: *x*-axis on both qubits and *x*-axis interacting control, square; (iii) Noise: N1 and N6 *z*-axis on each qubit; (iv) No distortion.
S_2q_IX-XI-XX_IZ-ZI_N1-N6_D	(i) Qubits: two; (ii) Control: *x*-axis on both qubits and *x*-axis interacting control, square; (iii) Noise: N1 and N6 *z*-axis on each qubit; (iv) Distortion.

The left column identifies each dataset in the respective QDataSet examples while the description column describes the profile of the square pulse datasets in terms of (i) number of qubits, (ii) axis of control and pulse wave-form (iii) axis and type of noise and (iv) whether distortion is present or absent.

### Future uses of the QDataSet

In this work, we have presented the QDataSet, a large-scale quantum dataset available for the development and benchmarking of quantum machine learning algorithms. The 52 datasets in the QDataSet comprise simulations of one- and two-qubit datasets in a variety of noise-free and noisy contexts together with a number of scenarios for exercising control. Large-scale datasets play an important role in classical machine learning development, often being designed and assembled precisely for the purpose of algorithm innovation. Despite its burgeoning status, QML lacks such datasets designed specifically to facilitate QML algorithm development. The QDataSet has been designed to address this need in the context of quantum control, tomography and noise spectroscopy, by providing a resource for cross-collaboration among machine learning practitioners, quantum information researchers and experimentalists working on applied quantum systems. In this paper we have also ventured a number of principles which we hope will assist producing large-scale datasets for QML, including specification of objectives, quantum data features, structuring, preprocessing. We set-out a number of key desiderata for quantum datasets in general. We also have aimed to provide sufficient background context across quantum theory, machine learning and noise spectroscopy for machine learning practitioners to treat the QDataSet as a point of entry into the field of QML. The QDataSet is sufficiently versatile to enable machine learning researchers to deploy their own domain expertise to design algorithms of direct use to experimental laboratories Table 9.

**Table 9:** An example of the types of quantum data features which may be included in a dedicated large-scale dataset for QML.

Item	Description
Quantum states	Description of states in computational basis, usually represented as vector or matrix (for *ρ*). May include initial and evolved (intermediate or final) states
Measurement operators	Measurement operators used to generate measurements, description of POVM.
Measurement distribution	Distribution of measurement outcome of measurement operators, either the individual measurement outcomes or some average (the QDataSet is an average over noise realisations).
Hamiltonians	Description of Hamiltonians, which may include system, drift, environment etc Hamiltonians. Hamiltonians should also include relevant control functions (if applicable).
Gates and operators	Descriptions of gate sequences (circuits) in terms of unitaries (or other operators). The representation of circuits will vary depending on the datasets and use case, but ideally quantum circuits should be represented in a way easily translatable across common quantum programming languages and integrable into common machine learning platforms (e.g. TensorFlow, PyTorch).
Noise	Description of noise, either via measurement statistics, known features of noise, device specifications.
Controls	Specification and description of the controls available to act on the quantum system.

The choice of such features will depend on the particular objectives in question. We include a range of quantum data in the QDataSet, including information about quantum states, measurement operators and measurement statistics, Hamiltonians and their corresponding gates, details of environmental noise and controls.

While designed specifically for problems in quantum control, tomography and noise mitigation, the scope for the application of the QDataSet in QML research is expansive. QML is an emerging cross-disciplinary field whose progression will benefit from the establishment of taxonomies and standardised practices to guide algorithm development. In this vein, we sketch below a number of proposals for the future use of the QDataSet, building upon principles upon which the QDataSet was designed, in order to foster the development of QML datasets and research practices.

1. *Algorithm development*. The primary research programme flowing from the QDataSet involves its use in the development of algorithms with direct applicability to quantum experimental and laboratory setups. As discussed above, the QDataSet has been designed to be versatile and of use across a range of use cases, such as quantum control, tomography, noise spectroscopy. In addition, its design enables machine learning practitioners to benchmark their algorithms. Future research involving the QDataSet could cover a systematic benchmarking of common types of classical machine learning algorithms for supervised and unsupervised learning. We also anticipate research programmes expanding upon greybox and hybrid models, using the QDataSet as a way to benchmark state of the art QML models.
2. *Quantum taxonomies*. While taxonomies within and across disciplines will differ and evolve, there is considerable scope for research programmes examining optimal taxonomic structuring of quantum datasets for QML. In this paper, we have outlined a proposed skeleton taxonomy that datasets for QML may wish to adopt or adapt, covering specification of objectives, ways in which data is described, identification of training (in-sample) and test (out-of-sample) data, data typing, structuring, completeness and visibility (see Supplementary Information for more detail). Further research in these directions could include expanding taxonomic classifications of QML in ways that connect with classical machine learning taxonomies, taking the QDataSet as an example. Doing so would facilitate greater cross-collaboration among computer scientists and quantum researchers by allowing researchers to easily transfer their domain expertise.
3. *Experimental interoperability*. An important factor in expanding the reach and impact of QML is the extent to which QML algorithms are directly applicable to solving problems in applied engineering settings. Ideally, QML results and architecture should be ‘platform agnostic’ - able to be applied to a wide variety of experimental systems, such as superconductor, transmon, trapped ion, photonic or quantum dot-based setups. Achieving interoperability across dynamically evolving technological landscapes is challenging for any discipline. For QML, the more that simulations within common platforms (such as those mentioned above) can easily integrate into each other and usefully simulate applied experiments, the greater the reach of algorithms trained using them. To the extent that the QDataSet can demonstrably be used across various platforms, algorithm design using it can assist these research imperatives.

We encourage participants in the quantum community to advance the development of dedicated quantum datasets for the benefit of QML and expect such efforts to contribute significantly to the advancement of the field and cross-disciplinary collaboration.

## Supplementary information

Supplementary Information

## Data Availability

The datasets are stored in an online repository and are accessible via links on the site. The largest of the datasets is over 500GB (compressed), the smallest being around 1.4GB (compressed). The QDataSet is provided subject to open-access MIT/CC licensing for researchers globally. The code used to generate the QDataSet is contained in the associated repository (see below), together with instructions for reproduction of the dataset. The QDataSet code requires Tensorflow > 2.0 along with a current Anaconda installation of Python 3. The code used to simulate the QDataSet is available via the Github repository^33^ (https://github.com/eperrier/QDataSet). A Jupyter notebook containing the code used for technical validation and verification of the datasets is available on this QDataSet Github repository.
