# Supplementary material for: QDataSet, quantum datasets for machine learning
Source: Sci Data. 2022 Sep 23;9:582. doi: 10.1038/s41597-022-01639-1 (PMC9508231; doi:10.1038/s41597-022-01639-1)
Supplement: Supplementary file 1 — Supplementary Information [file 41597_2022_1639_MOESM1_ESM.pdf]

QDataSet: Quantum Datasets for Machine Learning

Supplementary Information

August 11, 2022

Contents

1 Background Material **1**
1.1 Quantum postulates 1
1.1.1 State space 2
1.1.2 Operators and evolution 4
1.1.3 Composite systems 5
1.1.4 Measurement 5
1.1.5 Additional methodological concepts 6
1.1.6 Quantum metrics 6
1.1.7 Encoding data in quantum systems 8
1.2 QDataSet evolution 8
1.2.1 Evolution, Hamiltonians and control 8
1.2.2 Hamiltonians: drift, control, noise 10
1.2.3 QDataSet and Error-Correction 11

2 QML Objectives **12**
2.1 Overview 12
2.2 Large-Scale Data and Machine Learning 14
2.3 Characteristics of large-scale datasets 14
2.4 QML Datasets 19
2.5 QML and QC platforms 19
2.6 Quantum data in datasets 21

1 Background Material

1.1 Quantum postulates

Quantum information processing is characterised by constraints upon how information is represented and processed arising from the foundational postulates of quantum mechanics. Below we provide an overview of key elements of quantum postulates [1] and quantum information processing for classical machine learning practitioners solving optimisation problems for quantum engineering. In particular, we describe ways in which quantum data are typically

represented (such as via tensors), how quantum processes are usually expressed in common programming languages. The QDataSet is constructed to mimic conditions in laboratories and experiments where inputs and outputs to quantum systems are classical, such as via classically characterised controls (pulses, voltages) and measurement outcomes in the form of a classical probability distribution over observable outcomes of measurement. Actual quantum states, coherences and other characteristically quantum features of the system, while considered ontologically extant, are in effect reconstructions conditioned upon classical input and output data. In a machine learning context, this means that the encoding of quantum states, quantum processes (such as unitary evolution) represents the encoding of constraints upon how computation may evolve, rather than the input of actual quantum data. To this end, we follow the data generation protocols set out in [2] which we explicate below.

1.1.1 State space

Quantum systems are represented by (unit) state vectors within a complex-valued vector (Hilbert) space $\mathcal{H}$ whose dimensionality is determined according to the physics of the problem. There are various formalisms for representing such state vectors, but the predominant form in quantum information processing 'bra-ket' notation. In this notation, the state vector is represented as a 'ket' $|\psi\rangle \in \mathcal{H}$. Associated with a ket is a corresponding 'bra' notation $\langle\psi|$ which strictly speaking is a linear (one) form (or function) that acts on $|\psi\rangle$ such that $\langle\psi_1|\psi_2\rangle \in \mathbb{C}$ for two states $|\psi_1\rangle, |\psi_2\rangle$. The set of such linear forms forms a complementary vector space denoted the dual space. The majority of quantum information processing research to date has concentrated on qubits (quantum bits), being arbitrary two-level quantum systems (two-dimensional state spaces) of qubits with arbitrary state vectors with orthonormal bases $\{|0\rangle, |1\rangle\}$:

$$|\psi\rangle = a|0\rangle + b|1\rangle \tag{1}$$

Qubits are normalised such that they are unit vectors $\langle\psi|\psi\rangle = 1$ (that is $|a|^2 + |b|^2 = 1$), where $a, b \in \mathbb{C}$ are amplitudes for measuring outcomes of $|0\rangle, |1\rangle$ respectively (where $\langle\psi|\psi'\rangle$ denotes the inner product of quantum states $|\psi\rangle, |\psi'\rangle$). In density operator formalism, the system is described via a Hermitian positive semi-definite density operator ρ with trace unity acting on the state space of the system (such that if the system is in state ρ_i with probability p_i then $\rho = \sum_i p_i\rho_i$). Density operators are generalised operator-representations of probability distributions over quantum states with particular properties: all their eigenvalues have to be real, non-negative, sum to unity, inheriting the necessary constraints of a probability distribution.

In this work, we assume the standard orthonormal computational basis $\{|0\rangle, |1\rangle\}$ such that $\langle 1|0\rangle = \langle 0|1\rangle = 0$ and $\langle 1|1\rangle = \langle 0|0\rangle = 1$. Quantum states encode information of interest and use to optimisation problems. They are not directly observable, but rather their structure must be reconstructed from known information about the system. In machine learning contexts, quantum states may be used as inputs, constituent elements in intermediate computations or label (output) data. In the QDataSet, intermediate quantum states at any time step may be reconstructed using the intermediate Hamiltonians and unitaries for each example. The code repository for the QDataSet simulation provides further detail on how quantum state representations are used to generate the QDataSet [3]. Depending on machine learning architecture,

quantum states will usually be represented as matrices or tensors and may be used as inputs (for example, flattened), label data or as an intermediate input, such as in intermediate layers within a hybrid classical-quantum neural network (see [4,5]). For example, consider the matrix representation of eigenstates of a Pauli σ_z operator below:

$$\sigma_z = \begin{pmatrix} 1 & 0 \\ 0 & -1 \end{pmatrix} \tag{2}$$

In the computational basis, this operator has two eigenstates $|0\rangle, |1\rangle$ for eigenvalues $\lambda = 1, -1$:

$$|0\rangle = \begin{pmatrix} 1 \\ 0 \end{pmatrix} \quad \text{for} \quad \lambda = 1 \qquad |1\rangle = \begin{pmatrix} 0 \\ 1 \end{pmatrix} \quad \text{for} \quad \lambda = -1 \tag{3}$$

where we have adopted the formalism that the $\lambda = 1$ eigenstate is represented by $|0\rangle$ and the $\lambda = -1$ eigenstate is represented by $|1\rangle$ (our choice is consistent with Qutip - practitioners should check platforms they are using for the choice of representation). These eigenstates have a density operator representation as:

$$\rho_{\lambda=1} = |0\rangle\langle 0| \qquad \rho_{\lambda=-1} = |1\rangle\langle 1| \tag{4}$$

with matrix representations:

$$|0\rangle\langle 0| = \begin{pmatrix} 1 & 0 \\ 0 & 0 \end{pmatrix} \qquad |1\rangle\langle 1| = \begin{pmatrix} 0 & 0 \\ 0 & 1 \end{pmatrix} \tag{5}$$

For machine learning practitioners, one way to think about density operators is associating rows and columns with bra and ket vector representations:

$$\rho = a|0\rangle\langle 0| + b|0\rangle\langle 1| + c|1\rangle\langle 0| + d|1\rangle\langle 1| \doteq \begin{array}{c} \\ |0\rangle \\ |1\rangle \end{array} \begin{array}{c} \begin{array}{cc} \langle 0| & \langle 1| \end{array} \\ \begin{pmatrix} a & b \\ c & d \end{pmatrix} \end{array} \tag{6}$$

where $a, b, c, d \in \mathbb{C}$ are the complex-values amplitudes respective. Given $\rho = \sum_{p_i} \rho_i$, the diagonal elements a_n of the density matrix describe the probability p_n of the system residing in state ρ_n, that is

$$\rho_{nn} = a_n a_n^* = p_n \geq 0 \tag{7}$$

For pure states, the diagonal along the density matrix will only have one non-zero element (i.e. it will be 1) so that $\rho = \rho_i$. A mixed state will have multiple entries along the diagonal such that $0 \leq a_n < 1$. For example, the σ_z eigenvectors have the representation:

$$|0\rangle\langle 0| \doteq \begin{array}{c} \\ |0\rangle \\ |1\rangle \end{array} \begin{array}{c} \begin{array}{cc} \langle 0| & \langle 1| \end{array} \\ \begin{pmatrix} 1 & 0 \\ 0 & 0 \end{pmatrix} \end{array} \qquad |1\rangle\langle 1| \doteq \begin{array}{c} \\ |0\rangle \\ |1\rangle \end{array} \begin{array}{c} \begin{array}{cc} \langle 0| & \langle 1| \end{array} \\ \begin{pmatrix} 0 & 0 \\ 0 & 1 \end{pmatrix} \end{array} \tag{8}$$

Sometimes the density matrix representation of a state will be equivalent to the outer product of the state, but caution should be applied as this is not generally the case. Translating between the nomenclature and symbolism of quantum information to a more familiar matrix representation used in machine learning assists machine learning researchers to develop their algorithmic architecture. The QDataSet simulation code utilises state space representations of data and operations thereon in order to generate the output contained in the datasets themselves. To recover a quantum state $|\psi(t_j)\rangle = U(t_j)|\psi_0\rangle \approx \prod_i U_i^j |\psi_0\rangle$, one can simply apply the sequence U_i up to $i = j$ (note the order of application is such that $U_j ... U_0 |\psi\rangle$).

1.1.2 Operators and evolution

The Hamiltonian $H(t)$ of a system is primary means of mathematically characterising dynamics of quantum systems. Hamiltonians specify by the data-encoding process by which information is encoded into quantum states along with how the system evolves and how the quantum computation may be controlled. Unitary evolution itself is required to preserve quantum coherence and probability measures of systems (which give rise to the enhanced computational power of quantum systems). Algorithms trained using the QDataSet will usually involve modification to the controllable part of a system Hamiltonian in order to steer a system towards a desired state. This is to be understood more fully in terms of quantum state evolution.

Closed quantum systems (which we focus on in this paper for simplicity) evolve over time $\Delta t = t_1 - t_0$ via unitary transformations $U(\Delta t) = \mathcal{T}_+ \exp\left(-i\int_0^{\Delta t} H(t)dt\right)$ where $\mathcal{T}_+$ is the time-ordering operator (described below). As discussed below, given the difficulties in solving for time dependency, unitaries are typically approximated by time-independent sequences. The evolution of quantum states is characterised by such unitaries operating upon vectors that transform (transition) to other states. Intermediate quantum states $|\psi'\rangle$ may be represented as the result of applying unitary operators to initial states $|\psi\rangle$ such that $|\psi'\rangle = U(t)|\psi_0\rangle$ (or $\rho' = U(t)\rho_0 U(t)^\dagger$). Given initial quantum states, quantum state evolution can be represented entirely via operator dynamics and representations. There is a panoply of mathematical formalisms via which to understand operator dynamics, from representation theory, to operator algebras to category theory. From the perspective of a machine learning practitioners, operators will take the form of matrices or tensors in standard programming languages. It is worth noting that the operator acting on a quantum state ρ is a unitary $U(t)$ which is itself (in a closed quantum system) a function (or representation) of the Hamiltonian $H(t)$ governing the system dynamics. In practice unitaries are formed by exponentiating time-independent approximations of Hamiltonians and unitaries. These unitaries represent solutions to the time-dependent Schrödinger equation governing evolution:

$$i\hbar \frac{d|\psi(t)\rangle}{dt} = H(t)|\psi(t)\rangle \tag{9}$$

where $\hbar$ is set to unity for convenience and $H(t)$ represents the linear Hermitian operator (Hamiltonian) of the closed system. The dynamics of the quantum system are completely described by the Hamiltonian operator acting on the state $|\psi\rangle$ such that $|\psi(t)\rangle = U(t)|\psi(t=0)\rangle$. In density operator notation, such evolution is represented as $\rho(t) = U(t)\rho(t_0)U(t)^\dagger$. Typically

solving the continuous form of the Schrödinger equation is intractable or infeasible, so a discretised approximation as a discrete quantum circuit (where each gate U_i is run for a sufficiently small time-step Δt) is used (e.g. via Trotter-Suzuki decompositions).

1.1.3 Composite systems

States $|\psi\rangle$ in the Hilbert space may be composite systems, described as the tensor product of states spaces of the component physical systems, that is $|\psi\rangle = \otimes_i |\psi_i\rangle$. We also mention here the importance of open quantum systems where a total system Hamiltonian H can be decomposed as $H = H_S + H_E + H_I$, comprising a closed quantum system Hamiltonian H_S, an environment Hamiltonian H_E and an interaction Hamiltonian term H_I, which is typically how noise is modelled in quantum contexts. Open quantum systems are typically approximated using master equations to capture the dissipative effects of system/environment interaction. The dissipative nature of open quantum systems has parallels with the dissipative characteristics of neural networks (see [6]). Simulated data of open quantum systems can be generated in a number of packages, such as Qutip. For the QDataSet, we made a design decision to directly simulate the effects of coupling to dissipative environmental baths using more elementary Monte Carlo methods. The reason for this is that master equation formalism both requires a number of assumptions on the system (see [7, 8]) which may be difficult to apply to experimental setups. We also chose to manually engineer the effect of baths in order to minimise the theoretical barriers for classical machine learning practitioners using the QDataSet.

1.1.4 Measurement

Quantum measurements are framed as sets of measurement operators $\{M_m\}$, where m indexes the outcome of a measurement (e.g. an energy level or state indicator), i.e. an observable. The probability $p(m)$ of observable m upon measuring $|\psi\rangle$ is represented by such operators acting on the state such that $p(m) = \langle\psi|M_m^\dagger M_m|\psi\rangle$ (alternatively, $p(m) = \text{tr}(M_m^\dagger M_m \rho)$) with the post-measurement state $|\psi'\rangle$ given by:

$$|\psi'\rangle = \frac{M_m |\psi\rangle}{\sqrt{\langle\psi|M_m^\dagger M_m|\psi\rangle}} \tag{10}$$

The set of measurement operators $\sum_m M_m^\dagger M_m = I$ reflects the probabilistic nature of measurement outcomes. In more advanced treatments, positive-operator valued measure (POVM) formalism more fully describes the measurement statistics and post-measurement state of the system. For a POVM, we define a set of positive operators $\{E_m\} = \{M_m^\dagger M_m\}$ satisfying $\sum_m E_m = \mathbb{I}$ in a way that gives us a complete set of positive operators (such formalism being more general than simply relying on projection operators). As we are interested in probability distributions rather than individual probabilities from a single measurement, we calculate the probability distribution over outcomes via Born rule using the trace $p(E_i) = \text{Tr}(\rho E_i)$. We describe measurement procedures for the QDataSet in more detail below.

1.1.5 Additional methodological concepts

There are a number of other important concepts for classical machine learning practitioners to be aware of when using quantum datasets such as the QDataSet. We set out some of these: (a) *relative phase*, for a qubit system, amplitudes a and b differ by a relative phase if $a = \exp(i\theta)b, \theta \in \mathbb{R}$ (relative phase is an important concept as the relative phase encodes quantum coherences and is, together with basis encoding, a primary means of encoding data in quantum systems); (b) *entanglement*, composite states (known as EPR or Bell states), may be entangled, meaning that their measurement outcomes are necessarily correlated. For a two-qubit state:

$$|\psi\rangle = \frac{|00\rangle + |11\rangle}{\sqrt{2}} \tag{11}$$

a measurement outcome of 0 on the first qubit necessarily means that a measurement of the second qubit will result in the post-measurement state $|0\rangle$ also i.e. the measurement statistics of each qubit correlate. States that are entangled cannot be written as tensor products of component states i.e. $|\psi\rangle \neq |\psi_1\rangle |\psi_2\rangle$; (c) *expectation*, expectation values of an operator A (e.g. a measurement) can be written as $E(A) = \text{tr}(\rho A)$ (see below); (d) *mixed* and *pure* states, if the state of a quantum system is known exactly $|\psi\rangle$, i.e. where $\psi = |\psi\rangle \langle\psi|$ then it is denoted as a *pure state*, while where there is (epistemic) uncertainty about its state, it is a mixed state i.e. $\rho = \sum_i p_i \rho_i$ where $\text{tr}(\rho^2) < 1$ (as all $p_i < 1$); (e) *commutativity*, multiple measurements are performed on a system, the outcome will be order-dependent if the measurement operators corresponding to those measurements do not commute i.e if $[A, B] \neq 0$. This is distinct from the classical case; and (f) *no cloning*, quantum data cannot be copied, fundamentally distinguishing it from classical data. There are a range of other aspects of quantum systems that are relevant to the use of machine learning algorithms for solving optimisation problems which we pass over but which are relevant to research programmes using such algorithms, including the role of error-correcting codes (designed to limit or self-correct errors to achieve fault-tolerant quantum computing). While not the focus of the QDataSet, it is worth noting for machine learning practitioners that a distinction is usually drawn in the quantum information literature between *physical* and *logical* qubits. A physical qubit is a two-level physical quantum system. A logical qubit is itself an abstraction from a collection of physical qubits which in aggregate behave according to qubit parameters [9]. The QDataSet is generated on the assumption that each qubit is a logical qubit (which may or not equate to a single corresponding physical qubit). For more complex treatments (involving a multitude of physical qubits) in quantum control or quantum error correction, the underlying simulation code may be adapted accordingly.

1.1.6 Quantum metrics

Metrics play a central technical role in classical machine learning, fundamentally being the basis upon which machine learning algorithms update, via techniques such as backpropagation. Metrics for quantum information processing are related but distinct from their classical counterparts and understanding these differences is important for researchers applying classical machine learning algorithms to solve problems involving quantum data. As is commonplace

within machine learning, chosen metrics will differ depending on the objectives, optimisation strategies and datasets. For a classical bit string, there are a variety of classical information distance metrics used in general [1]. In more theoretical and advanced treatments, available metrics will depend upon the underlying structure of the problem (e.g. topology) (see [10] for a comprehensive discussion). Metrics used will depend also upon whether quantum states or operators are used as the comparators, though one can relatively easily translate between operator and state metrics. We outline a number of commonly used quantum metrics below and discuss their implementation in classical contexts, such as in loss functions. Note below we take license with the term metric as certain measures below, such as quantum relative entropy, do not (as with their classical counterparts) strictly constitute metrics as such.

1. *Hamming distance*, the number of places at which two bit strings are unequal. Hamming distance is important in error-correcting contexts and quantum communication [11].

2. *Trace distance* or *L*1-*Kolmogorov distance.* For two probability distributions $\{p_x\}, \{q_x\}$ we can construct the metric $D(p_x, q_x) = \frac{1}{2}\sum_x |p_x - q_x|$. In the quantum setting, for states represented by density matrices ρ, σ [1], their trace distance can be calculated as:

$$D(\rho, \sigma) = \frac{1}{2}\text{tr}|\rho - \sigma| \tag{12}$$

   where $|\rho| = \sqrt{\rho^\dagger \rho}$ is taken as the positive square root. Trace distance is a metric which is preserved under unitary transformations, thus is a widely used similarity metric in quantum information.

3. *Fidelity* is another common metric by which to assess state or operator similarity. Fidelity is given by $F(\rho, \sigma) = \text{tr}\sqrt{\rho^{1/2}\sigma\rho^{1/2}}$. It is among the most important metrics in quantum computing, being the measure by which quantum states or operators are measured. Fidelity can be interpreted as a metric by calculating the angle $\zeta = \arccos F(\rho, \sigma)$. Fidelity and trace distance are related via $D(\rho, \sigma) = \sqrt{1 - F(\rho, \sigma)^2}$.

4. *Quantum relative entropy*, is the quantum analogue of Shannon entropy. It is found given by $S(\rho) = -\text{tr}(\rho \log \rho)$ The quantum analogue of (binary) cross-entropy is in turn given by:

$$S(\rho||\sigma) = \text{tr}(\rho \log \rho) - \text{tr}(\rho \log \sigma) \tag{13}$$

   These measures provide a further basis for comparing for the output of algorithms to labelled data during training.

For most machine learning practitioners using the QDataSet, the entry point will be the application of known classical machine learning metrics. More advanced uses of the QDataSet may utilise quantum-specific metrics directly, for example, via reconstruction of quantum states from measurement statistics. Some use cases combine the use of classical and quantum metrics. For example, [2, 4] combine average operator fidelity with standard mean-squared error (MSE) into a measure denoted as ‘batch fidelity’. In those examples, the objective in question is to train a greybox algorithm to model certain control pulses needed to synthesise target unitaries.

The algorithms learn the particular control pulses which are applied to generators. While it is the extraction of control pulses which are of interest to experimentalists, the final output of the algorithm is a sequence of fidelities where the fidelity of generated (synthesised) unitaries is compared against the target (label) unitaries U_T. This sequence of fidelities is then compared against a vector of ones with the loss function set to minimise the MSE (distance) between the fidelity sequence and the label vector. In doing so, the algorithms are effectively trained to maximise fidelity (as fidelities ≈ 1 are desirable) yet do so using a hybrid approach. The QDataSet has been generated such that a combination of classical, quantum and hybrid metrics of divergence measures may be used in the training process.

1.1.7 Encoding data in quantum systems

Most quantum information optimisation problems involve information encoded in quantum systems, either by construction in an experiment involving quantum systems themselves, or via encoding exogenous or classical information into quantum systems (such as qubits) in order to leverage the benefits of quantum computation. Both approaches involve the input into quantum states in a process known as *state preparation.* The way in which data is encoded in quantum systems affects the performance and expressiveness many quantum algorithms [12]. Information is usually encoded using one of four standard encoding methods including [6]: (a) basis encoding, (b) amplitude encoding, (c) qsample encoding and (d) dynamic encoding. The first of these, *basis encoding*, is a technique that encodes classical information into quantum basis states. Usually, such procedures involve transformation of data into classical binary bit-strings $(x_1, ..., x_d), x_i \in \{0, 1\}$ then mapping each bit string to the quantum basis state of a set of qubits of a composite system. For example, for $x \in \mathbb{R}^N$, say a set of decimals, is converted into a d-dimensional bit string (e.g. $0.1 \to 00001..., -0.6 \to 11001..$) suitably normalised such that $x = \sum_k^d (1/2^k) x_k$. The sequence x is given a representation via $|\psi\rangle \propto |000001\,11001\rangle$ (see [6]). *Amplitude encoding* associates normalised classical information e.g. for an n-qubit system (with 2^n different possible (basis) states $|j\rangle$), a normalised classical sequence $x \in \mathbb{C}^{2^n}, \sum_k |x_k|^2 = 1$ (possibly with only real parts) with quantum amplitudes $x = (x_1, ..., x_{2^n})$ can be encoded as $|\psi_x\rangle = \sum_j^{2^n} x_j \,|j\rangle$. Other examples of sample-based encoding (e.g. *Qsample* and *dynamic* encoding are also relevant but not addressed here). From a classical machine learning perspective, such encoding regimes also enable both features and labels to be encoded into quantum systems.

1.2 QDataSet evolution

1.2.1 Evolution, Hamiltonians and control

The QDataSet is constructed on the basis of a typical quantum control [13, 14] problem, where it is assumed there exist a set of controls (such as pulses or voltages) with which the quantum system may be controlled or steered towards a target state (or unitary). In density matrix formalism, the Schrödinger equation is:

$$i\hbar \frac{d\rho}{dt} = [H(t), \rho(t)], \tag{14}$$

where the commutator $[A, B] = AB - BA$, $\hbar$ is Planck's constant (we can always choose the units such that $\hbar = 1$), and $H(t)$ is a Hermitian operator called the Hamiltonian. In physical systems, it corresponds to the total energy (sum of kinetic and potential energies) of the system under consideration. For a closed system (i.e. a noiseless isolated system with no interaction with the surrounding environment), it can be expressed in the general form:

$$H(t) = H_0(t) \doteq H_d(t) + H_{\text{ctrl}}(t). \tag{15}$$

$H_d(t)$ is called the drift Hamiltonian and corresponds to the natural evolution of the system in the absence of any control. The second term $H_{\text{ctrl}}(t)$ is called the control Hamiltonian and corresponds to the controlled external forces we apply to the system (such as electromagnetic pulses applied to an atom, or a magnetic field applied to an electron). The solution of the evolution equation at time $t = T$ is given by:

$$\rho(T) = U(T)\rho(0)U^\dagger(T), \tag{16}$$

where $\rho(0)$ is the initial state of the system, the unitary evolution matrix $U(t)$ is given by:

$$U(T) = \mathcal{T}_+ e^{-i\int_0^T H(t)dt} \tag{17}$$

$$\doteq \lim_{\Delta t \to 0} e^{-iH(T)\Delta t} \cdots e^{-iH(3\Delta t)\Delta t} e^{-iH(2\Delta t)\Delta t} e^{-iH(\Delta t)\Delta t} \tag{18}$$

The symbol $\mathcal{T}_+$ is called a time-ordering operator. The time-ordering is needed because in general the Hamiltonian is time-dependent and does not commute at different time instants (i.e. $[H(t_i), H(t_j)] \neq 0$). The second line is a time-independent approximation of the time-dependent form based on a Suzuki-Trotter decomposition [15,16] and Lie-Trotter formulation:

$$e^{-i(H_1+H_2)} = \lim_{n\to\infty} \left(e^{-i(H_1/n)} e^{-i(H_2/n)}\right)^n \tag{19}$$

which under certain conditions allows the time-varying Hamiltonian to be approximated by a piece-wise constant Hamiltonian. Here the time interval $[0, T]$ is divided into equal segments of length Δt. Moreover, we can achieve universal control (that is, the ability to synthesise all generators, by repeated application of the control Hamiltonian) via commutator terms that in turn allow us to generate the full generators of the corresponding Lie algebra. This can be shown via the approximation:

$$e^{-i[H1,H2]} = \lim_{n\to\infty} \left(e^{-iH_1/n} e^{-iH_2/n} e^{iH_1/n} e^{iH_2/n}\right)^n \tag{20}$$

For some machine learning applications, we are interested in minimising the number of controls that must be applied to a quantum system (thus minimising the resources required to control the system). In such cases, we may seek a minimal control algebra or gate set. The minimal number of Pauli operators required to achieve a complete control set of generators for synthesising an arbitrary unitary acting on an n dimensional Hilbert space is given by a bracket-generating set $\Delta \subseteq \mathfrak{su}(2^n)$ [4,17] which can be understood in more complex treatments in the context of Lie algebras and representation theory. Here $\mathfrak{su}(2^n)$ represents the Lie algebra corresponding to the Pauli group $\mathrm{SU}(2^n)$, the complete set of generators required to span the n dimensional

Hilbert space in the Pauli basis. Given Δ, we can reconstruct the full Pauli basis via the operation of the Lie bracket [18]. The QDataSet generators for one- and two-qubit systems are simply one and two (tensor-product) sets of Paulis respectively (i.e. not the minimal set Δ). For higher-dimensional problems, whether to restrict generators to those within Δ becomes a consideration for machine learning architectures (see literature on time-optimal quantum control such as Ref. [14]). These generators will typically be used as the tensors or matrices to which classical controls are applied within machine learning architectures.

As a special case if the Hamiltonian commute at different time instants then the evolution matrix can be simplified to:

$$U(T) = e^{-i\int_0^T H(t)dt}, \tag{21}$$

which also reduces to:

$$U(T) = e^{-iH(T)T}, \tag{22}$$

in the case of a time-independent Hamiltonian.

On the other hand when the system is open (i.e. interactions with the environment), then the Hamiltonian takes the form:

$$H(t) = H_0(t) + H_1(t) \doteq \underbrace{H_d(t) + H_{\text{ctrl}}(t)}_{H_0(t)} + \underbrace{H_{SE}(t) + H_E(t)}_{H_1(t)}. \tag{23}$$

$H_0(t)$ is defined as before to encompass the drift and control parts of the Hamiltonian. The new term $H_1(t)$ now consists of two terms: $H_{SE}(t)$ represents an interaction term with the environment, while $H_E(t)$ represents the free evolution of the environment in the absence of the system. In this case, the Hamiltonian controls the dynamics of both the system and environment combined in a highly non-trivial way. In other words, the state becomes the joint state between the system and environment. The combined system and environment then become closed. Modelling such open quantum systems is complex and challenging and is typically undertaken using a variety of stochastic master equations [7] or sophisticated noise spectroscopy. As detailed below, the QDataSet contains a variety of noise realisations for one and two qubit systems together with details of a recent novel operator [2] for characterising noise in quantum systems.

1.2.2 Hamiltonians: drift, control, noise

The QDataSet comprises datasets for one- and two- qubits systems evolving in the presence and absence of noise. The canonical forms of Hamiltonian from which those in the QDataSet are developed are given in [2]. In that work, a limited set of single-qubit systems subject to external environmental noise (baths) was used as input training data for a novel greybox machine learning alternative method for characterising noise. In this paper, the underlying simulation was modified to generate a greater variety of qubit-noise examples for the single qubit case. The simulation was then extended beyond that in [2] to generate examples for the two-qubit case (in the presence or absence of noise). As discussed above, the evolution of

closed and open quantum systems is described by Hamiltonian dynamics, which encode time-dependent functions into operators which are the generators of time-translations (operators) acting on quantum states. To summarise: the Hamiltonian comprises three elements: (i) a drift Hamiltonian $H_d(t)$, encoding the endogenous evolution of the quantum system; (ii) a control Hamiltonian $H_{\text{ctrl}}(t)$, encoding evolution due to the application of classical controls which may be applied to the quantum system to steer its evolution; and (iii) and an interaction (noise) Hamiltonian $H_1(t)$, encoding the effects of coupling of the quantum system to its environment, such as a decohering noise source (a bath). We express the Hamiltonians in the Pauli operator basis which forms a complete basis for our one- and two-qubit systems. Our control functions are represented as $f_\alpha(t)$ for $\alpha = x, y, z$ where the subscript indicates which generator the control applies to. Concretely, for example, σ_z control is denoted $f_z(t)\sigma_z$. In general, continuous time-dependent control formulations are difficult - or infeasible - to solve analytically, where solving here means finding a suitable representation of the control unitary:

$$U_{\text{ctrl}} = \mathcal{T}_+ e^{-i\int_0^T f_\alpha \sigma_\alpha / 2dt} \tag{24}$$

The simplest control functional form is fixed amplitude control [19] or what is also described as a *square pulse*, where a constant energy (expressed as amplitudes) is expressed for a discrete time-step Δt. Most controls are usually classical i.e. $f_\alpha(t) \in \mathbb{R}$. Other control waveforms include Gaussian. Moreover, quantum control in the QDataSet context has two primary imperatives. The first is the use of control in closed noise-free idealised quantum systems where the objective is the use of controls to steer the quantum system to some desired objective state. This is equivalent to the synthesis of quantum circuits (sequences of quantum gates) from the identity I to a target unitary U_T. The second is the use of controls in the presence of noise, where the quantum system is coupled to an environment that potentially decoheres the system. In this second case, ideally the controls are tailored to neutralise the effect of noise on the evolution of the quantum system - a process typically described by dynamic decoupling [20,21] (see for example Hahn echo or other examples). Crafting suitable controls to mitigate noise effects is challenging. One must properly time and select appropriate amplitudes for the application of controls to counteract decohering interference. Typically, it requires information about the noise spectrum itself, obtained using techniques from quantum noise spectroscopy [7]. It also requires an understanding of how control and noise signals convolve in the context of quantum evolution. Dealing with noise is a central imperative of quantum information processing and the engineering of quantum systems where the aim is to preserve and extend coherence times of quantum information and to correct for errors that arise during the evolution of quantum computational systems. To this end, a major stream of research in quantum information processing concerns quantum error-correcting codes (QEC) as means of encoding quantum information in a way that renders it robust to noise-induced errors and/or enables 'self-correction' of errors as a quantum system evolves.

1.2.3 QDataSet and Error-Correction

The QDataSet was generated for non-QEC encoded data. The reasoning behind this was that (i) specific error-correcting encodings differ considerably from case to case, whereas unencoded

quantum information is more prevalent in the experimental/laboratory setting; and (ii) quantum computational and NISQ devices are yet to reach the scale and prevalence necessary for practical testing of QEC at scale. The simulations in the QDataSet are based upon an alternative technique for quantum control in the presence of a variety of noise [2], where a greybox neural network is used to learn only those characteristics of the noise spectrum relevant to the application of controls (a comparatively simpler problem than seeking to determine the entire spectrum). In this context, the objective of the QDataSet is to enable algorithms to natively learn optimal error correction regimes from the data itself (rather than by encoding in a QEC) via inferring the types of countervailing controls (e.g. control pulses) that should be applied to minimise errors. In principle, the same type of machine-learning control architecture could also apply to QEC encoded data: the machine learning algorithms would in effect learn optimal quantum control conditioned on the data being encoded in an error-correcting code. Moreover, there is an emergent literature on using machine learning for QEC discover itself. For machine learning practitioners, the QDataSet thus provides a useful way to seek to apply advanced classical machine learning techniques to the challenging but important problem.

2 QML Objectives

2.1 Overview

Cross-disciplinary programmes focused on building quantum datasets for machine learning will benefit from a framework to categorise and classify the particular objectives of QML architectures and articulation of number of design principles relevant to the taxonomy of QML datasets. Designing large-scale datasets for QML requires an understanding of the objectives for which QML research is undertaken and the extent to which those objectives involve classical and/or quantum information processing. Following [22], the application of machine learning techniques to quantum information processing can be usefully parsed into a simple input / output and process taxonomy on the basis of whether information and computational processes are classical or quantum in nature. Here a process, input or output being 'quantum in nature' refers to the phenomenon by which the input or output data was generated, or by which the computational process occurs, is itself quantum in nature given that measurement outcomes are represented as classical datasets from which the existence of quantum states or processes is inferred. Quantum data encoded in logical qubits, for example in quantum states (superpositions or entangled), is different from classical data, in practical terms information about such quantum data arises by inference on measurement statistics whose outcomes are classical. This taxonomy can be usefully partitioned into four quadrants depending on the objectives of the QML task (to solve classical or quantum problems) and the techniques adopted (classical or quantum computational methods). Table (1) lists the various classical and quantum inputs according to this taxonomy.

1. *Classical machine learning for classical data.* The first quadrant covers the application of classical computational (machine learning) methods to solve classical problems, that is,

QML Taxonomy			
**QML Division**	**Inputs**	**Outputs**	**Process**
Classical ML	Classical	Classical	Classical
Applied classical ML	Quantum (Classical)	Classical (Quantum)	Classical
Quantum algorithms for classical problems	Classical	Classical	Quantum
Quantum algorithms for quantum problems	Quantum	Quantum	Quantum

Table 1: Quantum and classical machine learning table. Quantum machine learning covers four quadrants (listed in 'QML Division') which differ depending on whether the inputs, outputs or process is classical or quantum.

problems not involving data or processes of a quantum character.

2. *Classical machine learning for quantum data.* The second quadrant covers the application of classical computational and machine learning techniques to solving problems of a quantum character. Specifically, this subdivision of QML covers using standard machine learning techniques to solving problems specific to the theoretical or applied aspects of quantum computing, including optimal circuit synthesis [2, 4, 23], design of circuit architectures and so on. Either input or output data are quantum in nature, while the computational process by which optimisation, for example, occurs is itself classical.

3. *Quantum algorithms for classical optimisation.* The third quadrant of problem covers the application of quantum algorithmic techniques to solving classical problems. In this subdivision, algorithms are designed leveraging the unique characteristics of quantum computation, in a way that assist in optimising classical problems or solve certain classes of problems that may be intractable on a classical computer. Quantum algorithms are designed with machine learning characteristics, potentially utilising certain computational resources or processes unavailable when constrained to classical computation. Examples of such algorithms include variational quantum eigensolvers [24–27], quantum analogues of classical machine learning techniques (e.g. quantum PAC learning [28]) and hybrid quantum analogues of deep learning architectures (see Refs. [6, 29–31] for background).

4. *Quantum algorithms for quantum information processing.* The fourth quadrant covers the application of quantum algorithms to solve quantum problems, that is, problems whose input or output data is itself quantum in nature. This division covers the extensive field of quantum algorithm design, including the famous Grover and Shor algorithms [32–34].

The QDataSet fits within the second subdivision of QML, its primary use being envisaged as in the development of classical algorithms for optimisation problems of engineered quantum systems. Our focus on classical techniques applied to quantum data is deliberate: while advancements in quantum algorithms are both exciting and promising, the unavailability of a scalable fault-tolerant quantum computing system and limitations in hybrid NISQ devices mean that for the vast majority of experimental and laboratory use cases, the application of

machine learning is confined to the classical case. Secondly, as a major motivation of this paper is to provide an accessible basis for classical machine learning practitioners to enter the QML field, it makes sense to focus primarily on applying techniques from the classical domain to quantum data.

2.2 Large-Scale Data and Machine Learning

Classical machine learning has become one of the most rapidly advancing scientific disciplines globally with immense impact across applied and theoretical domains. The advancement and diversification of machine learning over the last two decades has been facilitated by the availability of large-scale datasets for use in the research and applied sciences. Large-scale datasets [35–37] have emerged in tandem with increasing computational power that has seen the velocity, volume and veracity of data increase [38, 39]. Such datasets have both been a catalyst for machine learning advancements and a consequence or outcome of increasing scope and intensity of data generation. The availability of large-scale datasets led to the evolution of data mining, applied engineering and even theoretical results in high energy physics [40].

An important lesson for QML is that developments within these fields have been facilitated using large-scale datasets in a number of ways. Large-scale datasets improve the trainability of machine learning algorithms by enabling finer-grained optimisations via commonplace methods such a backpropagation. This has particularly been true within the field of deep learning and neural networks [37], where large-scale datasets have enabled the development of deeper and richer algorithmic architectures able to model complex non-linearities and functional forms, in turn leading to drastic improvements and breakthroughs across domains such as image classification, natural language processing [41, 42] and time series analysis. With an abundance of data on which to train algorithms, new techniques such as regularisation and dimensionality reduction to address problems arising from large-scale datasets, including overfitting and complexity considerations, have in turn spurred novel innovations that have contributed to the advancement of the field. Large-scale datasets have also provided a *standardising* function by providing a common basis upon which algorithmic performance may be benchmarked and standardised. By providing standard benchmarks, large-scale datasets have enabled researchers to focus on important features of algorithmic architecture in the design of improvements to training regimes. Such datasets have also enabled the fostering of the field via competitive platforms such as Kaggle, where researchers compete to improve upon state of the art results.

2.3 Characteristics of large-scale datasets

Large-scale classical machine learning datasets share common structural and architectural characteristics designed to facilitate the objectives for which the datasets were compiled. There are a range of considerations including the specific objectives, the types of data to be stored, the degree of structuring of data (including whether highly structured or unstructured), the dimensionality of datasets, the extent of preprocessing of datasets required, data quality issues (such as missing, uncertain or incorrect data - an issue for example in quantum information processing contexts given sources of error and uncertainty), data imputation for missing datasets, visible or hidden data (e.g. whether data is direct or a feature constructed from other data), the number

Data Set Characteristics	
**Item**	**Description**
Objectives	Specification of the objectives for which the dataset was both created and to be used
Description	Sufficient description of data, representation and theoretical description
Training/test	Identification of training (in-sample) and test (out-of-sample) subsets of data
Data Types	Specification of the types of data and formats to be used
Structuring	Degree to which data is structured or unstructured
Dimensionality	The dimension of the datasets, dimensional reduction or kernel methods needed
Preprocessing	Extent to which preprocessing is required in dataset, covering transformations of data such as sparsification or decomposition
Data quality, consistency and completeness	Extent to which data is missing, uncertain or incorrect or noisy along with any necessary imputation methods
Visible v. hidden	Extent to which data points are 'direct' or 'indirect' (inferred) and whether imputations necessary

Table 2: Characteristics of large-scale datasets which can guide the generation of QML datasets and development of QML dataset taxonomies.

of data points, format, default tasks of the datasets, data temporality (how contemporaneous data is), control of datasets and access to data. Datasets are also structured depending on the machine learning algorithms for which they were developed, taking into account the types of objectives, loss functions, optimisers, development environment and programming languages of interest to researchers. Table (2) sets out a range of issues and desiderata in this regard.

Large-scale dataset characteristics affect the utility of the datasets in applied contexts. Such characteristics are relevant to the design of quantum datasets. Below we set out a number of principles used in the design of the QDataSet which we believe provide a useful taxonomy for the QML community to consider when generating data for use in machine learning-based problems. The aim of the proposed taxonomy for quantum datasets is to facilitate their interoperability across machine learning platforms (classical and quantum) and for use in optimisation for experimentalists and engineered quantum systems. While taxonomies and specific architectures will differ across domains, our proposed QDataSet taxonomy we believe will assist the QML and classical ML community to guide large-scale data generation towards principles of interoperability summarised in Table (2) and explained below:

1. *Objectives*. Quantum datasets, as with classical datasets, benefit from being constructed with particular objectives in mind. Most major classical large-scale datasets are compiled for specific objectives such as, for example, classification or regression tasks. In

a quantum setting, such objectives include quantum algorithm design, circuit synthesis, quantum control, tomography or measurement-based objectives (such as sampling). The QDataSet's objectives are to provide training data for use in the development of machine algorithms for controlled experimental and engineered quantum systems. This objective has informed the feature selection and structural design, such as inclusion of measurement statistics, Hamiltonian and unitary sequences and the various types of noise and distortion.

2. *Description.* Sufficiently describing the dataset, efficiently representing the data and providing theoretical context for how and why the datasets are so represented enhances the utility of datasets. Representation of data (its form, structure, data types and so on) affect the ease of use and uptake of datasets. For machine learning, optimal data representation is an important aspect of feature learning, representation learning [43]. In this paper, we go to some lengths to describe the various structural aspects of the QDataSet in order to facilitate its uptake by researchers in designing algorithms. We especially have set-out background information for machine learning practitioners who may be unfamiliar with quantum data in an effort to reduce barriers facing cross-disciplinary collaboration.

3. *Training and test sets.* Applied datasets for machine learning require training, validation and test sets in order to adequately train algorithms for objectives, such as quantum control. The design of quantum datasets in general, and the QDataSet in particular, has been informed by desirable properties of training sets. These include, for example: (i) *interoperability*, ensuring training set data can be adequately formatted for use in various programming languages (for example storing QDataSet data via matrices, vectors and tensors in Python); (ii) *generalisability*, preprocessing of datasets to improve generalisability of algorithmic results, especially to test or out-of-sample data [39] (in the QDataSet, we do this via providing a variety of noise-affected datasets); (iii) *feature smoothing* trained algorithms can often focus on information-rich yet small subspaces of data which, while informative for in-sample prediction, can lead to decreased generalisability across the majority of in- and out-of-sample data lacking such features. Feature smoothing is a technique to coarse-grain features so that less weight is put on rarer though information rich features in order to improve generalisation. In a quantum context, this may involve an iterative process of trial and error that trains datasets and seeks to identify relevant features; alternatively, it may involve using techniques from quantum information theory to classify regions of high and low information content e.g. via entropy measures.

4. *Data precision and type.* Data precision and data typing is an important consideration for quantum datasets, primarily to facilitate ease of interoperability between software and applied/hardware in the quantum space. Others considerations can include the degree of precision with which data should be presented. Ideally, quantum data for use in developing algorithms for application in experimental quantum control or measurement scenarios should allow flexibility of data precision to match instruments used in laboratories on a case-by-case basis. For example, the QDataSet precisification of noise degrees of freedom

(such as amplitude, mean and standard deviation) have been informed by collaborations with experimental groups.

5. *Structuring.* Data structuring, the degree to which data is structured according to taxonomies, is an important characteristic of classical datasets that affects their use and functionality. For quantum datasets, structuring encompasses the types of information that would be included and how that information is categorised. In the selection of real-world applicable datasets, researchers will have a range of choices of salient information to includes, including: theoretical details of the candidate Hamiltonians, details of the physical laboratory setting such as controls, exogenous parameters such as temperature (a significant environmental variable affecting quantum systems), noise or other disturbances; the characteristics of measurement devices and so on. Spectroscopic information, including details of the spectroscopy used, may also be included. What to include and not include will depend upon the particular uses cases and generality (or specificity) of the datasets. In each case, it makes sense for quantum datasets to contain as much useful information as possible such as about parameters, say exogenous environment parameters, or distortion information which may affect measurement devices. Doing so enables algorithms trained on quantum datasets to improve their performance and generalise better. Examples of such information in the QDataSet include details we have included regarding noise profiles and distortion simulations.

6. *Dimensionality.* The dimensionality of datasets is an important consideration. Large-dimensional datasets often require adaptation in order to facilitate algorithmic learning. This is especially in order to address the ubiquitous curse of dimensionality [44], where, as dimensions of datasets increase, algorithms may fail to converge, gradients may vanish or become stuck in barren plateaux. This may occur during the preprocessing stage, in-processing or during post-processing. Techniques such as principal component analysis [39], matrix factorisation [45], feature extraction together with algebraic techniques such as singular value decompositions are all motivated primarily to reduce the dimensionality and complexity of datasets, thereby minimising the hypothesis search space. Moreover, learning algorithms which can efficiently solve problems with sparse datasets often have computational advantages. Quantum data by its nature rapidly becomes higher-dimensional as the number of qubit or computations resources increases. Such vast search spaces present challenges for QML, such as the barren plateaux problems [46], the quantum analogue of the vanishing gradient problem in classical machine learning (albeit with differences arising due to exponentially-large search spaces).

7. *Preprocessing.* Datasets often require or benefit from preprocessing in order to ameliorate problems during training, such as vanishing gradients, bias or problems with convergence. Preprocessing data can include techniques such as sparsification [47] or smoothing or other strategies. For example, for quantum circuit synthesis, ensuring training data samples are drawn from across the Hilbert space of interest rather than limited to subspaces can assist with generalisation (see Ref. [4] for a geometric example). In such cases, quantum dataset

preparation may benefit from preprocessing to address sparsity concerns (see Ref. [48] for examples and for classical analogues of vanishing gradients [49]).

8. *Visibility.* Classical machine learning is often concerned with extraction - or development - of features. In many forms of classical machine learning, such as those using kernel methods, or deep learning, features of importance to optimal performance of an algorithm may need to be inferred from the data. Quantum datasets in many ways face such challenges from the outset as quantum data can never be directly observed, rather it must be inferred from measurement statistics. When constructing quantum datasets, the extent to which such inferred (as distinct from directly observed) data will be an important choice. In the QDataSet, we have chosen to include a range of such 'hidden' or 'inferred' data to assist practitioners with use of the dataset, including the intermediate forms of Hamiltonian, unitaries and other data that is not itself directly accessible but is a by-product of our simulation (accessible via intermediate layers).

Studying features of particular datasets and their use in classical contexts assists in extracting desirable features for large-scale quantum datasets. ImageNet is one of the leading research projects and database architectures for images [36, 37, 50]. The dataset is one of the most widely cited and important datasets in the development of machine learning, especially image-classification algorithms using convolutional, hierarchical and other deep-learning based neural networks. The evolution of ImageNet and its use within machine learning disciplines provides a useful guide and comparison for the development of QML datasets in general. ImageNet comprises two main components: (i) a public semi-structured dataset of images together with (ii) an annual competition and workshop. The dataset provides a 'ground truth' standardised set of images against which to train categorical image classification algorithms. The competition and workshop provided and continue to provide an important institutional practice driving machine learning development. While beyond the scope of this paper, the development of QML would arguably be considerably assisted by the availability of QML-focused competitions akin to those commonplace within the classical machine learning community. Such competitive frameworks would motivate and drive the development of scalable and generalisable QML algorithms. As is also evident from classical machine learning, competitive formats are also a useful way for laboratories, companies or other projects to leverage the expertise of the diverse machine learning community.

Another example from the machine learning community which can inform the development of QML is Kaggle, a leading online platform for machine learning-based competitions. Kaggle runs competitions where competitors are provided with prediction tasks, training sets and constraints upon the type of algorithm design (such as resource use and so on). Competitors then develop models aiming to optimise a measure of success, such as a standard machine learning metric of accuracy, AUC or some other measure [51]. The open competitive nature of Kaggle is designed to crowd-source solutions and expertise to problems in machine learning and data science. A 'quantum Kaggle' would be of considerable benefit to the QML community by providing a platform through which to spur collaborative and competitive development of quantum algorithms.

2.4 QML Datasets

While quantum datasets for machine learning (quantum and classical) are neither as prevalent nor as ubiquitous as those in the classical realm, there are some examples in the literature of quantum or hybrid quantum-classical datasets generated for use in machine learning contexts. QML datasets can be categorised into: (1) *general quantum datasets* produced for purposes other than QML, such as quantum datasets in quantum chemistry of other fields, which can be preprocessed or used as training data in QML contexts. Such datasets are not specifically produced for the purposes of QML per se; (2) dedicated *QML-specific quantum datasets*, generated and structured for the purposes of QML. This second category mainly consists of quantum datasets for use in classical or hybrid machine learning contexts. Quantum datasets currently available tend towards one or other of these classifications, though there is overlap, for example, with quantum datasets designed for use in machine learning which are nevertheless highly domain-specific. Examples include quantum chemistry datasets for use in deep tensor neural networks [52], datasets for learning spectral properties of molecular systems [53–56] and for solid-state physics [57–60].

A recent example is provided by the dedicated quantum chemistry datasets known as the *QM7-X dataset* [61], an expansive dataset of physiochemical properties for several million equilibrium and non-equilibrium structures of small organic molecules. The QM7-X dataset spans regions of chemical compound space and was generated to provide a basis for machine-learning assisted design of molecules with specific properties. The dataset builds upon previous iterations of QM-series and related quantum chemistry datasets [55,62,63]. Structurally, the dataset combines global (molecular) properties and local (atomic) information, including ground state quantities (spectra and moments) along with response quantities (related to polarisation and dispersion). The dataset is highly domain-specific and represents a salient example of a dataset designed to spur machine-learning driven research within a particular field.

2.5 QML and QC platforms

The use of quantum datasets in machine learning has been facilitated over the last several years by a surge in quantum programming and languages and platforms for both QML and quantum computing generally. While such platforms are dynamic and changing, it is important that quantum datasets for machine learning be constructed to be as interoperable with platforms in the quantum and classical machine learning community. Generators of large-scale quantum datasets should be cognisant of how their data can be (more easily) used in such platforms below and also how their datasets can be designed in ways that facilitates their ease of use within common machine learning languages, such as TensorFlow, PyTorch and across languages, such as Python, C#, Julia and others. The QDataSet has been specifically designed in relatively elementary Python packages such as Numpy in order to facilitate its use across the machine learning community, but also in a way that we hope makes it useable and clearly understandable by the quantum engineering community. We deliberately selected Python as the language of choice within which to build the QDataSet simulation given its status as the leading classical programming language of choice for machine learning. It also is a language

adopted across many of the quantum platforms above. We built the QDataSet using Numpy to produce datasets as transferable as possible (rather than, for example, in Qutip). A familiarity with the emerging quantum programming and QML ecosystem is useful for the design of quantum datasets. We set out a few examples of leading quantum programming platforms below.

Qutip [8] is a leading quantum simulation and algorithmic package in Python used for open quantum systems' simulation. The package, while not developed specifically for QML, is widely used for hybrid quantum-classical systems' research. Inputs to Qutip algorithms are Numpy-based vectors, tensors and matrices used to represent density matrices, quantum states and operators. Qutip permits a wide range of simulations to be run and data to be generated, including for state preparation, control and drift Hamiltonians, pulse sequences and noise modelling. As discussed below, the QDataSet, which was built in Python using primarily the Numpy package, but was verified using Qutip. *Q#* is Microsoft's primary open-source programming language for quantum algorithm design and execution. The platform comprises a number of libraries, simulators and a software development kit (QDK). *Quantum Tensorflow* (QTF) [64] is a hybrid quantum-classical version of Google's leading open-source machine learning Tensorflow platform. QTF is constructed to enable the synthesis of classical and quantum algorithmic machine learning, for example classically parameterised quantum circuits, variational quantum circuits and eigensolvers, quantum convolutional neural networks and other quantum analogues of classical machine learning architectures. QTF follows Tensorflow's overall machine learning structure and data taxonomy. Input data is usually in the form of tensors. QTF's in-platform datasets vary depending on use case, but the platform primarily draws upon classical datasets for hybrid use cases (quantum computation applied to solving classical optimisation tasks). For simulated quantum-native data, QTF draws upon *Cirq*, Google's open source framework for programming quantum computers [65]. Cirq is focused on providing a software library for research into and simulations of quantum circuits, the idea being to develop quantum algorithms in Cirq that can be run on quantum computers and simulators.

Strawberry Fields [66] is an open-source QML and quantum algorithm programming platform developed by Xanadu for photonic quantum computing. *Qiskit* is another open source software development kit for quantum circuits, control and applications on quantum and hybrid computers [67]. Qiskit is based on an open source quantum assembly language (QASM) standardised abstraction of quantum circuits. Other platforms enabling the integration of quantum datasets and QML algorithms (either quantum or classical) include those available via IBM's Quantum Experience. The QDataSet has been designed to for interoperability across most of these platforms. Practically speaking, this means that researchers can select dataset features of interest, such as tensors of quantum states, Hamiltonians, unitary operators (gates) or even noise information and integrate as datasets for use in algorithms designed using platforms above. Similarly, machine learning researchers should find the form of data relatively familiar to typical datasets in machine learning, where information is encoded in tensors, lists or matrices. Examples of using similar datasets in customised TensorFlow machine learning models can be found in various sources [2, 4].

2.6 Quantum data in datasets

An important aspect of quantum dataset design is the decision regarding what quantum information to include in the dataset. In this section, we list types of quantum data which may be included in large-scale quantum datasets. By *quantum data*, we refer to data generated or characterising quantum systems or processes. Quantum data may comprise a range of different properties, features or characteristics of quantum systems, the environment around quantum systems. It may comprise data and information abstracted into a particular representation or form, such as circuit gates, algebraic formulations, codes etc or more physical forms, such as statistics or read-outs from measurement devices. For QML datasets, it is useful to ensure that quantum data is sufficient for classical machine learning researchers to understand and for integrating quantum data into their algorithmic techniques. For example, a classically parameterised quantum circuit, as common throughout the QML literature, would typically include data or tensors of the relevant parameters, the operators related to such parameters (such as generators) and the quantum states (vectors or density operators) upon which the circuit acts.

References

[1] M.A. Nielsen and I.L. Chuang. *Quantum Computation and Quantum Information: 10th Anniversary Edition.* Cambridge University Press, 2010.

[2] Akram Youssry, Gerardo A. Paz-Silva, and Christopher Ferrie. Characterization and control of open quantum systems beyond quantum noise spectroscopy. *npj Quantum Information*, 6(1):1–13, December 2020.

[3] Elija Perrier, Akram Youssry, and Chris Ferrie. QDataSet: Quantum Datasets for Machine Learning. *Cloudstor Repository* `https://cloudstor.aarnet.edu.au/plus/s/rxYKXBS7Tq0kB8o`, August 2021.

[4] Elija Perrier, Dacheng Tao, and Chris Ferrie. Quantum geometric machine learning for quantum circuits and control. *New Journal of Physics*, 22(10):103056, October 2020.

[5] Akram Youssry, Robert J. Chapman, Alberto Peruzzo, Christopher Ferrie, and Marco Tomamichel. Modeling and control of a reconfigurable photonic circuit using deep learning. *Quantum Science and Technology*, 5(2):025001, January 2020.

[6] Maria Schuld and Francesco Petruccione. Supervised Learning with Quantum Computers. In Maria Schuld and Francesco Petruccione, editors, *Supervised Learning with Quantum Computers*, Quantum Science and Technology, pages 1–19. Springer International Publishing, Cham, 2018.

[7] H. M. Wiseman and G. J. Milburn. *Quantum Measurement and Control.* Cambridge University Press, URL, 2010.

[8] J. R. Johansson, P. D. Nation, and Franco Nori. QuTiP 2: A Python framework for the dynamics of open quantum systems. *Computer Physics Communications*, 184(4):1234–1240, April 2013.

[9] Bilal Shaw, Mark M. Wilde, Ognyan Oreshkov, Isaac Kremsky, and Daniel A. Lidar. Encoding one logical qubit into six physical qubits. *Physical Review A*, 78(1):012337, July 2008. Publisher: American Physical Society.

[10] John Watrous. *Theory of Quantum Information.* Cambridge University Press, 2018.

[11] João Fernando Doriguello and Ashley Montanaro. Quantum sketching protocols for Hamming distance and beyond. *Physical Review A*, 99(6):062331, June 2019. Publisher: American Physical Society.

[12] Maria Schuld. Supervised quantum machine learning models are kernel methods. *arXiv:2101.11020 [quant-ph, stat]*, Apr 2021. arXiv: 2101.11020.

[13] Yu. L. Sachkov. Control theory on lie groups. *Journal of Mathematical Sciences*, 156(3):381, January 2009.

[14] D. D'Alessandro. *Introduction to Quantum Control and Dynamics*. Chapman & Hall/CRC Applied Mathematics & Nonlinear Science. CRC Press, 2007.

[15] Masuo Suzuki. Quantum Monte Carlo methods — recent developments. *Physica A: Statistical Mechanics and its Applications*, 194(1):432–449, March 1993.

[16] Andrew M. Childs, Yuan Su, Minh C. Tran, Nathan Wiebe, and Shuchen Zhu. A theory of trotter error. *Physical Review X*, 11(1):011020, Feb 2021. arXiv: 1912.08854.

[17] R. Montgomery, American Mathematical Society, P. Landweber, M. Loss, T.S. Ratiu, and J.T. Stafford. *A Tour of Subriemannian Geometries, Their Geodesics, and Applications*. Mathematical surveys and monographs. American Mathematical Society, 2002.

[18] Michael Swaddle, Lyle Noakes, Harry Smallbone, Liam Salter, and Jingbo Wang. Generating three-qubit quantum circuits with neural networks. *Physics Letters A*, 381(39):3391–3395, October 2017.

[19] Heinz Schättler and Urszula Ledzewicz. *Geometric Optimal Control: Theory, Methods and Examples*. Springer Science & Business Media, June 2012.

[20] Riddhi Swaroop Gupta and Michael J. Biercuk. Machine Learning for Predictive Estimation of Qubit Dynamics Subject to Dephasing. *Physical Review Applied*, 9(6):064042, June 2018.

[21] Sandeep Mavadia, Virginia Frey, Jarrah Sastrawan, Stephen Dona, and Michael J. Biercuk. Prediction and real-time compensation of qubit decoherence via machine learning. *Nature Communications*, 8, January 2017.

[22] Esma Aïmeur, Gilles Brassard, and Sébastien Gambs. Machine Learning in a Quantum World. In Luc Lamontagne and Mario Marchand, editors, *Advances in Artificial Intelligence*, Lecture Notes in Computer Science, pages 431–442, Berlin, Heidelberg, 2006. Springer.

[23] Gregory Riviello, Katharine Moore Tibbetts, Constantin Brif, Ruixing Long, Re-Bing Wu, Tak-San Ho, and Herschel Rabitz. Searching for quantum optimal controls under severe constraints. *Physical Review A*, 91(4):043401, April 2015.

[24] Alberto Peruzzo, Jarrod McClean, Peter Shadbolt, Man-Hong Yung, Xiao-Qi Zhou, Peter J. Love, Alán Aspuru-Guzik, and Jeremy L. O'Brien. A variational eigenvalue solver on a photonic quantum processor. *Nature Communications*, 5:4213, July 2014. _eprint: 1304.3061.

[25] Zhi-Cheng Yang, Armin Rahmani, Alireza Shabani, Hartmut Neven, and Claudio Chamon. Optimizing Variational Quantum Algorithms Using Pontryagin's Minimum Principle. *Physical Review X*, 7(2):021027, May 2017.

[26] Dave Wecker, Matthew B. Hastings, and Matthias Troyer. Progress towards practical quantum variational algorithms. *Physical Review A*, 92(4):042303, October 2015. Publisher: American Physical Society.

[27] Jarrod R. McClean, Jonathan Romero, Ryan Babbush, and Alán Aspuru-Guzik. The theory of variational hybrid quantum-classical algorithms. *New Journal of Physics*, 18(2):023023, February 2016. _eprint: 1509.04279.

[28] Seth Lloyd, Masoud Mohseni, and Patrick Rebentrost. Quantum principal component analysis. *Nature Physics*, 10(9):631–633, September 2014.

[29] Guillaume Verdon, Jason Pye, and Michael Broughton. A Universal Training Algorithm for Quantum Deep Learning. Preprint at `http://arxiv.org/abs/1806.09729`, 2018.

[30] Thomas Vidick and John Watrous. Quantum Proofs. *Foundations and Trends in Theoretical Computer Science*, 11(1-2):1–215, 2016.

[31] Zheng An and D. L. Zhou. Deep reinforcement learning for quantum gate control. *EPL (Europhysics Letters)*, 126(6):60002, Jul 2019.

[32] Lov K. Grover. Quantum Mechanics Helps in Searching for a Needle in a Haystack. *Physical Review Letters*, 79(2):325–328, July 1997. Publisher: American Physical Society.

[33] Gilles Brassard and Peter Hoyer. An Exact Quantum Polynomial-Time Algorithm for Simon's Problem. *Proceedings of the Fifth Israeli Symposium on Theory of Computing and Systems*, pages 12–23, 1997. arXiv: quant-ph/9704027.

[34] Peter W. Shor. Polynomial-Time Algorithms for Prime Factorization and Discrete Logarithms on a Quantum Computer. *SIAM Journal on Computing*, 26(5):1484–1509, 1997. arXiv: quant-ph/9508027.

[35] Yann LeCun, Corinna Cortes, and Christopher J C Burges. The MNIST database of handwritten digits. Dataset at `https://yann.lecun.com/exdb/mnist`, 1998.

[36] Jia Deng, Wei Dong, Richard Socher, Li-Jia Li, Kai Li, and Li Fei-Fei. ImageNet: A large-scale hierarchical image database. In *2009 IEEE Conference on Computer Vision and Pattern Recognition*, pages 248–255, June 2009. ISSN: 1063-6919.

[37] I. Goodfellow, Y. Bengio, and A. Courville. *Deep Learning*. Adaptive Computation and Machine Learning series. MIT Press, 2016.

[38] Rob Kitchin and Gavin McArdle. What makes Big Data, Big Data? Exploring the ontological characteristics of 26 datasets. *Big Data & Society*, 3(1):2053951716631130, June 2016. Publisher: SAGE Publications Ltd.

[39] T. Hastie, R. Tibshirani, and J. Friedman. *The Elements of Statistical Learning: Data Mining, Inference, and Prediction*. Springer Series in Statistics. Springer New York, New York, 2013.

[40] Kim Albertsson, Piero Altoe, Dustin Anderson, Michael Andrews, Juan Pedro Araque Espinosa, Adam Aurisano, Laurent Basara, Adrian Bevan, Wahid Bhimji, Daniele Bonacorsi, Paolo Calafiura, Mario Campanelli, Louis Capps, Federico Carminati, Stefano Carrazza, Taylor Childers, Elias Coniavitis, Kyle Cranmer, Claire David, Douglas Davis, Javier Duarte, Martin Erdmann, Jonas Eschle, Amir Farbin, Matthew Feickert, Nuno Filipe Castro, Conor Fitzpatrick, Michele Floris, Alessandra Forti, Jordi Garra-Tico, Jochen Gemmler, Maria Girone, Paul Glaysher, Sergei Gleyzer, Vladimir Gligorov, Tobias Golling, Jonas Graw, Lindsey Gray, Dick Greenwood, Thomas Hacker, John Harvey, Benedikt Hegner, Lukas Heinrich, Ben Hooberman, Johannes Junggeburth, Michael Kagan, Meghan Kane, Konstantin Kanishchev, Przemys\law Karpiński, Zahari Kassabov, Gaurav Kaul, Dorian Kcira, Thomas Keck, Alexei Klimentov, Jim Kowalkowski, Luke Kreczko, Alexander Kurepin, Rob Kutschke, Valentin Kuznetsov, Nicolas Köhler, Igor Lakomov, Kevin Lannon, Mario Lassnig, Antonio Limosani, Gilles Louppe, Aashrita Mangu, Pere Mato, Helge Meinhard, Dario Menasce, Lorenzo Moneta, Seth Moortgat, Meenakshi Narain, Mark Neubauer, Harvey Newman, Hans Pabst, Michela Paganini, Manfred Paulini, Gabriel Perdue, Uzziel Perez, Attilio Picazio, Jim Pivarski, Harrison Prosper, Fernanda Psihas, Alexander Radovic, Ryan Reece, Aurelius Rinkevicius, Eduardo Rodrigues, Jamal Rorie, David Rousseau, Aaron Sauers, Steven Schramm, Ariel Schwartzman, Horst Severini, Paul Seyfert, Filip Siroky, Konstantin Skazytkin, Mike Sokoloff, Graeme Stewart, Bob Stienen, Ian Stockdale, Giles Strong, Savannah Thais, Karen Tomko, Eli Upfal, Emanuele Usai, Andrey Ustyuzhanin, Martin Vala, Sofia Vallecorsa, Justin Vasel, Mauro Verzetti, Xavier Vilasís-Cardona, Jean-Roch Vlimant, Ilija Vukotic, Sean-Jiun Wang, Gordon Watts, Michael Williams, Wenjing Wu, Stefan Wunsch, and Omar Zapata. Machine Learning in High Energy Physics Community White Paper. *Journal of Physics: Conference Series*, 1085:022008, September 2018. Publisher: IOP Publishing.

[41] Jacob Devlin, Ming-Wei Chang, Kenton Lee, and Kristina Toutanova. BERT: Pre-training of Deep Bidirectional Transformers for Language Understanding. *arXiv:1810.04805 [cs]*, May 2019. arXiv: 1810.04805.

[42] Tom B. Brown, Benjamin Mann, Nick Ryder, Melanie Subbiah, Jared Kaplan, Prafulla Dhariwal, Arvind Neelakantan, Pranav Shyam, Girish Sastry, Amanda Askell, Sandhini Agarwal, Ariel Herbert-Voss, Gretchen Krueger, Tom Henighan, Rewon Child, Aditya Ramesh, Daniel M. Ziegler, Jeffrey Wu, Clemens Winter, Christopher Hesse, Mark Chen, Eric Sigler, Mateusz Litwin, Scott Gray, Benjamin Chess, Jack Clark, Christopher Berner, Sam McCandlish, Alec Radford, Ilya Sutskever, and Dario Amodei. Language Models are Few-Shot Learners. *arXiv:2005.14165 [cs]*, July 2020. arXiv: 2005.14165.

[43] William L. Hamilton, Rex Ying, and Jure Leskovec. Representation Learning on Graphs: Methods and Applications. *arXiv:1709.05584 [cs]*, September 2017. arXiv: 1709.05584.

[44] Richard Bellman. Dynamic Programming and Lagrange Multipliers. *Proceedings of the National Academy of Sciences of the United States of America*, 42(10):767–769, October 1956.

[45] Carlo Ciliberto, Mark Herbster, Alessandro Davide Ialongo, Massimiliano Pontil, Andrea Rocchetto, Simone Severini, and Leonard Wossnig. Quantum machine learning: a classical perspective. *Proceedings of the Royal Society A: Mathematical, Physical and Engineering Sciences*, 474(2209):20170551, January 2018. Publisher: Royal Society.

[46] Jarrod R. McClean, Sergio Boixo, Vadim N. Smelyanskiy, Ryan Babbush, and Hartmut Neven. Barren plateaus in quantum neural network training landscapes. *Nature Communications*, 9:4812, November 2018. _eprint: 1803.11173.

[47] Saiprasad Ravishankar, Bihan Wen, and Yoram Bresler. Online Sparsifying Transform Learning—Part I: Algorithms. *IEEE Journal of Selected Topics in Signal Processing*, 9(4):625–636, June 2015. Conference Name: IEEE Journal of Selected Topics in Signal Processing.

[48] Carlos Ortiz Marrero, Mária Kieferová, and Nathan Wiebe. Entanglement Induced Barren Plateaus. *arXiv:2010.15968 [quant-ph]*, October 2020. arXiv: 2010.15968.

[49] Razvan Pascanu, Tomas Mikolov, and Yoshua Bengio. On the difficulty of training recurrent neural networks. In *Proceedings of the 30th International Conference on International Conference on Machine Learning - Volume 28*, ICML'13, pages III–1310–III–1318, Atlanta, GA, USA, June 2013. JMLR.org.

[50] Olga Russakovsky, Jia Deng, Hao Su, Jonathan Krause, Sanjeev Satheesh, Sean Ma, Zhiheng Huang, Andrej Karpathy, Aditya Khosla, Michael Bernstein, Alexander C. Berg, and Li Fei-Fei. ImageNet Large Scale Visual Recognition Challenge. *International Journal of Computer Vision*, 115(3):211–252, December 2015.

[51] Casper Solheim Bojer and Jens Peder Meldgaard. Kaggle forecasting competitions: An overlooked learning opportunity. *International Journal of Forecasting*, 37(2):587–603, April 2021.

[52] Kristof T. Schütt, Farhad Arbabzadah, Stefan Chmiela, Klaus R. Müller, and Alexandre Tkatchenko. Quantum-chemical insights from deep tensor neural networks. *Nature Communications*, 8(1):13890, January 2017. Number: 1 Publisher: Nature Publishing Group.

[53] Stefan Chmiela, Alexandre Tkatchenko, Huziel E. Sauceda, Igor Poltavsky, Kristof T. Schütt, and Klaus-Robert Müller. Machine learning of accurate energy-conserving molecular force fields. *Science Advances*, 3(5):e1603015, May 2017. Publisher: American Association for the Advancement of Science Section: Research Article.

[54] Raghunathan Ramakrishnan, Mia Hartmann, Enrico Tapavicza, and O. Anatole von Lilienfeld. Electronic spectra from TDDFT and machine learning in chemical space. *The Journal of Chemical Physics*, 143(8):084111, August 2015. Publisher: American Institute of Physics.

[55] Matthias Rupp, Alexandre Tkatchenko, Klaus-Robert Müller, and O. Anatole von Lilienfeld. Fast and Accurate Modeling of Molecular Atomization Energies with Machine Learning. *Physical Review Letters*, 108(5):058301, January 2012. Publisher: American Physical Society.

[56] Albert P. Bartók, Michael J. Gillan, Frederick R. Manby, and Gábor Csányi. Machine-learning approach for one- and two-body corrections to density functional theory: Applications to molecular and condensed water. *Physical Review B*, 88(5):054104, August 2013. Publisher: American Physical Society.

[57] Christopher Sutton, Luca M. Ghiringhelli, Takenori Yamamoto, Yury Lysogorskiy, Lars Blumenthal, Thomas Hammerschmidt, Jacek R. Golebiowski, Xiangyue Liu, Angelo Ziletti, and Matthias Scheffler. Crowd-sourcing materials-science challenges with the NOMAD 2018 Kaggle competition. *npj Computational Materials*, 5(1):1–11, November 2019. Number: 1 Publisher: Nature Publishing Group.

[58] Chandramouli Nyshadham, Matthias Rupp, Brayden Bekker, Alexander V. Shapeev, Tim Mueller, Conrad W. Rosenbrock, Gábor Csányi, David W. Wingate, and Gus L. W. Hart. Machine-learned multi-system surrogate models for materials prediction. *npj Computational Materials*, 5(1):1–6, April 2019. Number: 1 Publisher: Nature Publishing Group.

[59] Wojciech J. Szlachta, Albert P. Bartók, and Gábor Csányi. Accuracy and transferability of Gaussian approximation potential models for tungsten. *Physical Review B*, 90(10):104108, September 2014. Publisher: American Physical Society.

[60] Felix A. Faber, Alexander Lindmaa, O. Anatole von Lilienfeld, and Rickard Armiento. Machine Learning Energies of 2 Million Elpasolite \$(AB{C}_{2}{D}_{6})\$ Crystals. *Physical Review Letters*, 117(13):135502, September 2016. Publisher: American Physical Society.

[61] Johannes Hoja, Leonardo Medrano Sandonas, Brian G. Ernst, Alvaro Vazquez-Mayagoitia, Robert A. DiStasio Jr., and Alexandre Tkatchenko. QM7-X, a comprehensive dataset of quantum-mechanical properties spanning the chemical space of small organic molecules. *Scientific Data*, 8(1):43, February 2021.

[62] Lars Ruddigkeit, Ruud van Deursen, Lorenz C. Blum, and Jean-Louis Reymond. Enumeration of 166 Billion Organic Small Molecules in the Chemical Universe Database GDB-17. *Journal of Chemical Information and Modeling*, 52(11):2864–2875, November 2012. Publisher: American Chemical Society.

[63] Justin S. Smith, Roman Zubatyuk, Benjamin Nebgen, Nicholas Lubbers, Kipton Barros, Adrian E. Roitberg, Olexandr Isayev, and Sergei Tretiak. The ANI-1ccx and ANI-1x data sets, coupled-cluster and density functional theory properties for molecules. *Scientific Data*, 7(1):134, May 2020. Number: 1 Publisher: Nature Publishing Group.

[64] Michael Broughton, Guillaume Verdon, Trevor McCourt, Antonio J. Martinez, Jae Hyeon Yoo, Sergei V. Isakov, Philip Massey, Murphy Yuezhen Niu, Ramin Halavati, Evan Peters,

Martin Leib, Andrea Skolik, Michael Streif, David Von Dollen, Jarrod R. McClean, Sergio Boixo, Dave Bacon, Alan K. Ho, Hartmut Neven, and Masoud Mohseni. TensorFlow Quantum: A Software Framework for Quantum Machine Learning. *arXiv:2003.02989 [cond-mat, physics:quant-ph]*, March 2020. arXiv: 2003.02989.

[65] Cirq Developers. Cirq, May 2021.

[66] Nathan Killoran, Josh Izaac, Nicolás Quesada, Ville Bergholm, Matthew Amy, and Christian Weedbrook. Strawberry Fields: A Software Platform for Photonic Quantum Computing. *Quantum*, 3:129, March 2019. arXiv: 1804.03159.

[67] Gadi Aleksandrowicz, Thomas Alexander, Panagiotis Barkoutsos, Luciano Bello, Yael Ben-Haim, David Bucher, Francisco Jose Cabrera-Hernández, Jorge Carballo-Franquis, Adrian Chen, Chun-Fu Chen, Jerry M. Chow, Antonio D. Córcoles-Gonzales, Abigail J. Cross, Andrew Cross, Juan Cruz-Benito, Chris Culver, Salvador De La Puente González, Enrique De La Torre, Delton Ding, Eugene Dumitrescu, Ivan Duran, Pieter Eendebak, Mark Everitt, Ismael Faro Sertage, Albert Frisch, Andreas Fuhrer, Jay Gambetta, Borja Godoy Gago, Juan Gomez-Mosquera, Donny Greenberg, Ikko Hamamura, Vojtech Havlicek, Joe Hellmers, Łukasz Herok, Hiroshi Horii, Shaohan Hu, Takashi Imamichi, Toshinari Itoko, Ali Javadi-Abhari, Naoki Kanazawa, Anton Karazeev, Kevin Krsulich, Peng Liu, Yang Luh, Yunho Maeng, Manoel Marques, Francisco Jose Martín-Fernández, Douglas T. McClure, David McKay, Srujan Meesala, Antonio Mezzacapo, Nikolaj Moll, Diego Moreda Rodríguez, Giacomo Nannicini, Paul Nation, Pauline Ollitrault, Lee James O'Riordan, Hanhee Paik, Jesús Pérez, Anna Phan, Marco Pistoia, Viktor Prutyanov, Max Reuter, Julia Rice, Abdón Rodríguez Davila, Raymond Harry Putra Rudy, Mingi Ryu, Ninad Sathaye, Chris Schnabel, Eddie Schoute, Kanav Setia, Yunong Shi, Adenilton Silva, Yukio Siraichi, Seyon Sivarajah, John A. Smolin, Mathias Soeken, Hitomi Takahashi, Ivano Tavernelli, Charles Taylor, Pete Taylour, Kenso Trabing, Matthew Treinish, Wes Turner, Desiree Vogt-Lee, Christophe Vuillot, Jonathan A. Wildstrom, Jessica Wilson, Erick Winston, Christopher Wood, Stephen Wood, Stefan Wörner, Ismail Yunus Akhalwaya, and Christa Zoufal. Qiskit: An Open-source Framework for Quantum Computing, January 2019. Language: en.
